# TCR clonotyping of paired blood and tonsillar T-follicular cells reveals the mixed origin of CD38^high^ Tfrs

**DOI:** 10.3389/fimmu.2026.1947623

**Published:** 2026-10-09

**Authors:** Sophiya R. Sokolova, Vladimir A. Grigoriants, Irina A. Shagina, Ekaterina V. Barsova, Maria A. Nomerovskaya, Jamilya Z. Khalifaeva, Alexander V. Gurov, Olga V. Britanova, Ivan V. Zvyagin, Dmitriy M. Chudakov, Irina L. Grigorova

**Affiliations:** 1 Institute of Translational Medicine, Pirogov Russian National Research Medical University, Moscow, Russia; 2 Center for Bio and Medical Technology, Moscow, Russia; 3 Genomics of Adaptive Immunity Department, Shemyakin and Ovchinnikov Institute of Bioorganic Chemistry, Moscow, Russia; 4 Department of Otholaryngology, Pirogov Russian National Research Medical University, Moscow, Russia; 5 Department of Otholaryngology, City Clinical Hospital No. 1 named after N.I. Pirogov, Moscow, Russia; 6 Sverzhevsky Scientific Research Clinical Institute of Otorhinolaryngology, Moscow, Russia

**Keywords:** circulating Tfr, GC Tfh, human tonsils, induced Tfr, natural Tfr, non-follicular Tregs, scRNA-seq, TCR clonotyping

## Abstract

Circulating T follicular regulatory (cTfr) cells are measured as blood biomarkers after vaccination, in autoimmune disease (e.g., lupus, rheumatoid arthritis) and cancer immunotherapy, yet whether they originate from natural Tregs (yielding nTfr) or germinal center T follicular helper (GC Tfh) cells (yielding iTfr) remains unknown. This distinction is critical for interpreting cTfr expansion as Treg-derived regulation versus Tfh-derived negative feedback. To characterize tonsillar follicular T cell heterogeneity and resolve the origins of cTfr cells we applied single-cell multiomics and bulk T cell receptor sequencing (TCRβ-seq) analysis of matched human tonsil and blood. Tonsillar nTfr and iTfr cells distribute across shared transcriptional states, with *CD38* expression distinguishing iTfr from nTfr in most contexts. However, within a transitional cluster positioned between activated Tfh and Tfr cells, nTfr-like cells also upregulated CD38, indicating that its specificity for iTfr is not absolute. Peripheral blood CD38^-^ cTfr cells shared clonotypes exclusively with non-follicular Tregs and lacked overlap with GC Tfh, indicating predominantly Treg origin. CD38^high^ cTfr cells captured a mixed population: some shared clonotypes with tonsillar GC Tfh (iTfr), while others shared clonotypes with non-follicular Tregs (nTfr). These findings establish that accurate discrimination of iTfr versus nTfr within the CD38^high^ compartment requires TCR clonal reference to matched tissue.

## 1 Introduction

Follicular helper T (Tfh) and follicular regulatory T (Tfr) cells have garnered significant interest due to their dual importance in physiology and disease. On the one hand, these cells play critical roles in supporting B cell responses, including antibody production, class-switching, and regulation of autoreactive B cells (1, 2). On the other hand, their dysregulation has been linked to multiple pathological conditions, such as allergic reactions and autoimmunity (3, 4). Furthermore, the interplay between Tfh and Tfr has been implicated in the regulation of the tumor microenvironment across various cancers (5, 6) and in other disorders, including transplantation complications and chronic viral infection (2, 7, 8).

Tfh cells play an important role in the early plasmablast response and are essential for the formation and maintenance of germinal centers (GC), where they facilitate GC B cell class-switching, selection, and differentiation into memory B cells and plasma cells (9–13). Tfh also produce CXCL13 and play a role in the formation of tertiary lymphoid structures (14, 15). In contrast, Tfr cells regulate humoral immunity by suppressing autoreactive B cell clones (16–18), regulating the magnitude of foreign-antigen-specific B cell response and plasma cell formation (19–22), affecting class-switching (23–26), and fine-tuning B cell affinity maturation (27–29).

Given these critical functions, circulating Tfh (cTfh) and Tfr (cTfr) cells are increasingly monitored in human blood as biomarkers of humoral immune response in vaccination and disease (30–34). However, interpreting these blood-based studies is complicated by the heterogeneous developmental origins of Tfr cells. Unlike Tfh cells, which arise from conventional CD4^+^ T cells in response to foreign antigens, Tfr cells can originate from three distinct precursors: thymic natural Tregs (nTfr), GC Tfh cells (iTfr), or peripheral Tregs (pTfr) (20, 21, 35–37). These populations are further distinguished by their antigen specificity and function: nTfr cells are thought to be predominantly self-antigen-specific and function to restrain autoantigen-acquiring and -presenting GC B cells (29, 38), whereas pTfr and iTfr cells are likely directed against foreign antigens and may contribute to regulation and timely resolution of the foreign-antigen-specific GC response (37). Despite these functional distinctions, differentiating these subsets, particularly in the circulation, remains a major challenge.

A previous study reported high levels of CD38 on iTfr cells from pediatric tonsils, suggesting this marker could distinguish iTfr from nTfr (35). However, these analyses relied on separately sorted subpopulations, leaving it unclear whether CD38 reliably differentiates these subsets within the heterogeneous follicular T cell pool in secondary lymphoid organs. Moreover, it remains uncertain whether observations made in pediatric tonsils are applicable to adult human tonsils, given that GC activity and Treg pool composition are age-dependent (39, 40). Finally, whether GC Tfh-derived iTfr cells can enter the circulation, and if so, whether they can be distinguished from nTfr cells by CD38 expression, remains unclear.

To address this, we combined single-cell transcriptomics and TCR repertoire analysis of matched adult human tonsil and peripheral blood to resolve the origins of circulating Tfr cells. Clonotypic sharing indicated that circulating CD38^-^ Tfr cells derive exclusively from nTregs, whereas CD38^high^ cTfr cells represent a mixed population of both iTfr and nTfr. These findings suggest a mixed developmental origin for circulating Tfr cells and underscore the importance of tracking both CD38 expression and TCR clonal relationships in blood-based immune monitoring.

## 2 Methods

### 2.1 Patient cohort

A complete list of human donors included in the study is provided in **Supplementary Table S1**. Fresh palatine tonsils were obtained from adult patients (20–58 years) undergoing tonsillectomy for recurrent tonsillitis without acute infection. Peripheral blood (18 mL) for TCRβ profiling was collected from the same patients. Additional blood samples from healthy donors (23–60 years) were used for follicular T cell immunophenotyping. The study was approved by the Local Ethics Committee of Pirogov RNRMU (Protocol No. 240, 23.05.2024). All participants gave written informed consent.

### 2.2 Tissue and blood processing

Tonsils were processed within 2 h of surgery. Organ processing was done according to the protocol from (41). Tissues were minced and passed through 70 µm cell strainers. Peripheral blood was collected in K3EDTA tubes. Tonsillar and blood mononuclear cells were isolated by Ficoll density gradient centrifugation as previously described (42). Cell counting was performed using trypan blue exclusion.

### 2.3 Phenotypic analysis and intracellular staining

Mononuclear cells (2 × 10^6^ per sample) were stained with fluorescent antibody panels (see **Supplementary Table S2**) as previously described (41). For intracellular staining, cells were fixed/permeabilized using True-Nuclear™ Transcription Factor Buffer Set (Biolegend, San Diego, CA, USA) and stained overnight at +4 °C with anti-BCL6, anti-FOXP3, and anti-KI67 antibodies. Flow cytometry was performed on FACS Aria™ III (BD Biosciences, San Jose, CA, USA) and Cytek Aurora spectral flow cytometer (Cytek Biosciences, Fremont, CA, USA). Data were analyzed with FlowJo v10.8.1 (FlowJo, LLC., Ashland, OR, USA); t-SNE was used for cell subset visualization. Graphs were created in GraphPad Prism v8.4.3 (GraphPad Software, LLC., Boston, MA, USA).

### 2.4 Fluorescence-activated cell sorting

Mononuclear cells (20 × 10^6^) from matched blood and tonsils were washed with Sorting buffer (0.5% FBS in PBS), Fc-blocked (10 min, +4 °C), stained with antibody mix (**Supplementary Table S2**) for 30 min on ice, washed twice, and resuspended in Sorting buffer. Sorting was performed on a FACS Aria™ III. (BD Biosciences, San Jose, CA, USA). A 70 µm nozzle (60 psi) was used for circulating cells; a 100 µm nozzle (20 psi) for tonsillar cells. Purity mode: 4-way with purity mask enabled. Purity of sorted populations exceeded 96%.

For bulk TCR-seq, circulating follicular T cell subpopulations from blood were sorted in four technical replicates directly into UniSoRT Buffer (MiLaboratory, Boston, MA, USA) (43). Tonsillar GC Tfh and non-follicular Tregs were isolated into complete RPMI-1640 medium (four replicates each). For single-cell RNA-seq, tonsillar CD4^+^ CD19^-^ CXCR5^+^ cells were enriched for CXCR5^+^ CD25^high^ Tfh/Tfr cells (ratios 8:1 and 5:1 for donors 1 and 2). Cells were collected into complete RPMI-1640 medium and kept on ice before downstream processing. Details on the number of cells sorted for bulk RNA-seq are provided in **Supplementary Table S3**.

### 2.5 Bulk TCRβ cDNA library preparation and sequencing

Sorted tonsillar GC Tfh and non-follicular Tregs were pelleted and resuspended in RLT buffer (Qiagen, Hilden, North Rhine-Westphalia, Germany). RNA was extracted using phenol-chloroform (44). UMI-labeled TCRβ cDNA libraries were constructed from blood and tonsil samples using a Human Multiplex RNA TCR Kit (MiLaboratory, Boston, MA, USA) per manufacturer’s instructions. cDNA was generated with Hiscript III Reverse Transcriptase (Nanjing Vazyme Biotech Co., Ltd, Nanjing, Jiangsu, China), and amplicons with a Multiplex PCR Kit (Qiagen, Hilden, North Rhine-Westphalia, Germany). Libraries were pooled and sequenced on SURFSeq 5000 (Shenzhen GeneMind Biosciences Co., Ltd, Shenzhen, Guangdong, China) with 150 + 150 bp paired-end reads. Details of the bulk RNA-seq data are provided in **Supplementary Table S3**.

### 2.6 10× sc 5`RNA and paired TCRαβ sequencing library preparation

Sorted tonsillar CD4^+^CD19^-^CXCR5^+^ cells were resuspended in DPBS with 0.04% BSA (New England Biolabs, Ipswich, MA, USA). Single-cell suspensions (target ~20,000 cells per donor) were loaded onto a Chromium X controller (10× Genomics, Pleasanton, CA, USA). Gene expression and TCR libraries were constructed using Chromium Next GEM Single Cell 5’ Reagent Kit v2 Dual Index (10× Genomics, Pleasanton, CA, USA). Sequencing was performed on DNBSEQ-G400 (MGI Tech Co., Ltd., Shenzhen, Guangdong, China) at depths of 20,000 reads/cell (gene expression) and 5,000 reads/cell (TCR libraries). Details of the 10× single-cell data are provided in **Supplementary Table S4**.

### 2.7 Bulk TCRβ-seq data processing

A total of 50 × 10^6^ TCRβ reads (1.6 × 10^4^–7.3 × 10^6^ per library) were obtained from 48 libraries (6 populations × 4 replicates × 2 donors). Unique UMI-labeled TCRβ cDNA molecules (8.6 × 10^5^ total; 150–1.8 × 10^5^ per library) were assembled into clonotypes using MiXCR v4.6 (45), *milab-human-rna-tcr-umi-multiplex* preset. Functional TCRβ clonotypes with read count ≥4 were retained. Frequencies were calculated as UMI count/total UMI count. D50 diversity index was calculated using Python 3.10, repseq library (46).

### 2.8 TCRβ repertoire downsampling

Three downsampling strategies were used depending on the analysis context: (1) top clonotype selection with tail mixing: clonotypes ranked by UMI count, top N retained, ties at the boundary resolved by random subset selection; (2) random clonotype selection: unique clonotypes (V + J + CDR3nt) randomly drawn from the full repertoire without weighting by clonotype abundance; (3) random UMI downsampling: the repertoire was downsampled to a target number of randomly chosen UMI-labeled cDNA molecules. In all cases, clonotype frequencies were recalculated within the downsampled set, and a fixed random seed was used.

In figure legends, “clonotype downsampling” refers to top clonotype selection (option 1) unless stated otherwise.

### 2.9 TCRβ repertoires clonotype sharing

Clonotypes were considered shared between two repertoires when they had identical TCRβ V and J genes and CDR3 nucleotide sequence. Sharing was quantified as the percentage of shared clonotypes within the repertoire of interest or as the number of shared clonotypes, as indicated in the figure legends. Sharing was assessed for each donor independently. Where a population was compared with both tonsillar bulk repertoires, clonotypes detected in GC Tfh were counted as GC Tfh-shared and the n.f. Treg category was restricted to clonotypes not detected in GC Tfh. Repertoires of unequal size were downsampled as indicated in the figure legends.

### 2.10 Single-cell RNA and TCR sequencing analysis

Raw 10× Genomics Chromium 5’ scRNA-seq and V(D)J scTCR-seq data were processed using Cell Ranger (v9.0.0) (10× Genomics, Pleasanton, CA, USA) (47). Filtered gene expression matrices were analyzed in Seurat (v5) (48). Cells containing <1,000 UMIs or >6,500 UMIs or >8% mitochondrial genes were removed. Gene expression was normalized using SCTransform for each donor’s matrix separately, after which the two donor’s datasets were merged. PCA was performed on 2,800 shared variable features, identified with SelectIntegrationFeatures. The first 10 PCs were used for FindNeighbors and UMAP. Clustering was performed with the Louvain algorithm at resolution 1.3, yielding 20 clusters. Differentially expressed genes (DEGs) were identified per cluster using FindAllMarkers (Wilcoxon rank-sum, min.pct = 0.2). A hierarchical dendrogram was built using BuildClusterTree with the top 3,000 unique DEGs as features (48). For dot plot heatmaps, mean log-normalized expression was z-scored per gene across clusters.

### 2.11 Single-cell cluster connectivity

Inter-cluster connectivity was assessed using the minimum spanning tree (MST) step of Slingshot (49) applied to cluster centroids in 3D UMAP space. Pre-Tfh cluster 7 was used as a root, and the resulting MST was validated independently for each donor. Consensus edges were defined as connections present in both donors. The resulting consensus tree was projected onto 2D UMAP with edges connecting cluster centroids.

### 2.12 Single-cell TCRβ intersection analyses

For scTCR intersection with bulk TCR-seq populations, single-cell clonotypes were defined by TCRβ V + J + CDR3nt identity. Cells were classified by sharing with bulk GC Tfh or n.f. Tregs only. For iTfr/nTfr differential expression, cells within the Tfr pool whose clonotype matched bulk GC Tfh were classified as iTfr; those matching only n.f. Tregs were classified as nTfr.

### 2.13 TABLO atlas single-cell data analysis

Single-cell RNA and TCR data from adult donors 7–10 of the TABLO human T cell atlas (50) were obtained from Zenodo (per-donor matrices with paired αβ TCR: 13119615; full-transcriptome Seurat objects: 18868813). From the original TABLO annotation, we selected CD161^+^ and memory Tregs, as well as Tfh and GC Tfh cells (corresponding to TABLO clusters 14, 17, 19, 21, 22, 26, 31, 33, and 38), yielding 344,187 cells. These cells were projected onto the 20-cluster follicular T cell reference generated in this study using FindTransferAnchors and MapQuery (Seurat v5, LogNormalize, dims = 1:10) based on 1,248 shared variable features. Cells with a prediction score ≥0.7 were retained for downstream analysis (60.0% of projected cells).

To focus on the Tfr compartment, we restricted the projected TABLO cells to those mapping onto reference Tfr clusters 8, 10, 16, and 0, resulting in a pool of 48,579 cells. Two reference repertoires were defined outside this pool: (1) n.f. Tregs, consisting of TABLO cluster 27 (naïve Tregs) with no detectable *CXCR5* expression (30,471 cells); and (2) GC Tfh cells, defined as cells mapping onto reference cluster 14 (13,212 cells). To assign Tfr cells as iTfr or nTfr, we used paired αβ TCR clones defined by identical V gene, J gene, and CDR3 nucleotide sequence. Cells in the Tfr pool whose clones matched the GC Tfh repertoire were classified as iTfr (including 25 cells of 3,437 that also matched the n.f. Treg repertoire); cells matching only the n.f. Treg repertoire were classified as nTfr.

### 2.14 Statistical analysis

Group comparisons were performed using two-sided Mann–Whitney U tests for unstratified analyses, with Benjamini–Hochberg correction applied for multiple comparisons where applicable. For donor-stratified comparisons (e.g., analyses of cell subsets nested within individual donors), we used the van Elteren test, a stratified extension of the Wilcoxon rank-sum test that accounts for donor-level clustering. Analyses were implemented in Python (v3.10; pandas, NumPy, SciPy, matplotlib, seaborn) and R (v4.3; ggplot2, ComplexHeatmap). Flow cytometry data analyses were performed using GraphPad Prism v8.4.3 (GraphPad Software, LLC., Boston, MA, USA). All statistical data is included in Supplementary **Data Sheet 1.xlxs**. All experimental and statistical details are provided in the figure legends.

## 3 Results

### 3.1 Identification of follicular T cell subsets in human tonsils

To delineate the distinguishing features of GC-derived iTfr and Treg-derived nTfr cells among all follicular T cell subsets, and to ascertain their frequency in the circulation, we undertook a comparative assessment of Tf cells isolated from tonsils and peripheral blood. Human tonsils represent a site of ongoing GC and Tfh responses. We therefore reasoned that follicular T cells (Tf) generated at this site would constitute a substantial fraction of the circulating memory Tf cell pool. Furthermore, this locale would enable more extensive accumulation of GC Tfh-derived iTfr cells, facilitating their direct comparison with nTfr cells and other follicular T cell subsets.

To conduct a comparative analysis of Tfh and Tfr cells in human tonsils and blood, PBMCs were isolated from two adult donors with chronic tonsillitis before tonsillectomy. Circulating Tfh and Tfr cells were sorted from blood for bulk profiling of TCRβ CDR3 repertoires (**Figure 1A**). To investigate the precursors of follicular T cell subsets, particularly GC Tfh and Treg cells, we sorted CD4^+^CD25^-/int^CXCR5^high^PD1^high^ GC Tfh and CD4^+^CXCR5^-^CD25^high^ Treg cells (non-follicular, n.f.) from human tonsils in multiple replicates (**Figure 1A**) using the gating strategy indicated in **Figure 1B** (see **Supplementary Table S3**). The GC Tfh gate was validated by BCL6 expression (**Supplementary Figure 1A**) (51) and contained fewer than 0.5% FOXP3+ cells (**Supplementary Figure 1B**). Over 90% of cells in the n.f. Treg gate were FOXP3^+^ (**Supplementary Figure 1C**). We then performed bulk TCRβ repertoire sequencing of sorted GC Tfh and n.f. Tregs. TCRβ clonotypes were defined based on the unique V and J gene segments and CDR3 nucleotide sequences (V + J + CDR3nt). Only a small fraction of the GC Tfh and n.f. Treg repertoires was shared between the two populations, in contrast to the high reproducibility between technical replicates within each population (**Supplementary Figures 1D–F**). We therefore adopted a classification strategy to maximize Treg population specificity. Specifically, the GC Tfh repertoire was defined to include all clonotypes detected in the GC Tfh fraction, whereas the n.f. Treg repertoire was restricted to clonotypes exclusively detected in that subset.

**Figure 1:**
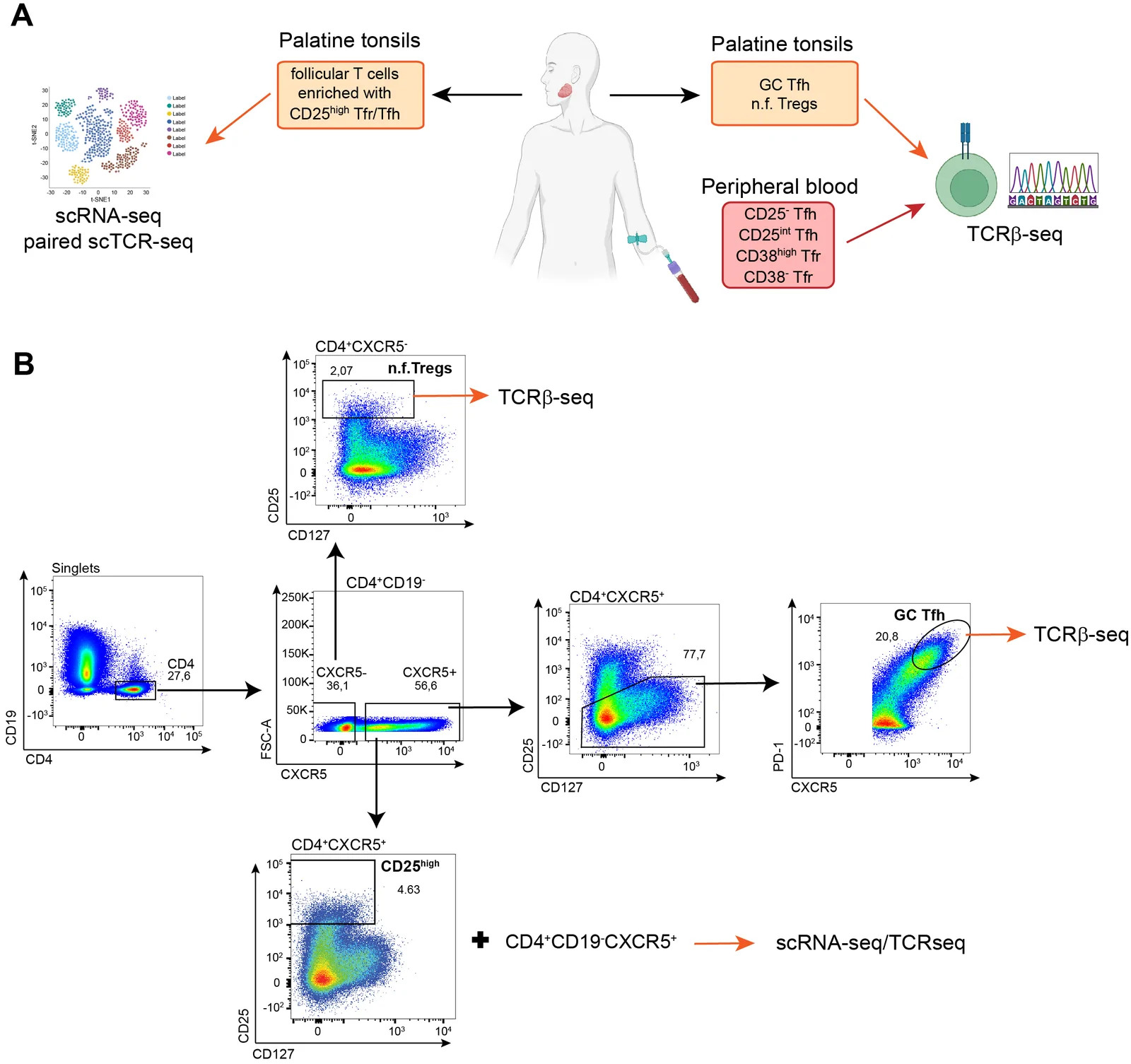
Experimental design and gating strategy for sorting tonsillar n.f. Tregs, GC Tfh, and follicular T cells. **(A)** Experimental design of the study. Mononuclear cells were isolated from matched human peripheral blood and palatine tonsil samples. Tonsillar germinal center (GC) Tfh and non-follicular (n.f.) Tregs were FACS-sorted in multiple replicates for TCR-seq, while total follicular (CD4^+^ CXCR5^+^) T cells enriched for CD25^high^ cells were isolated for 10× 5’ single-cell RNA-seq and paired TCR-seq. In parallel, CD25^−^ Tfh, CD25^int^ Tfh, CD38^high^ Tfr, and CD38^−^ Tfr from peripheral blood were collected in replicates for TCR-seq and comparative bioinformatics analysis with tonsillar T cell subsets. **(B)** Gating strategy for sorting tonsillar n.f. Tregs, GC Tfh, and combined follicular T cells enriched for CD25^high^ cells. Panel **(A)** created with BioRender.com.

To identify follicular T cell subsets in human tonsils, we performed paired single-cell RNA-seq/TCR-seq analysis of all sorted CD4^+^CD19^−^CXCR5^+^ follicular T cells combined with CD4^+^CD19^−^CXCR5^+^CD25^high^ cells from the same donors (at 8:1 and 5:1 ratios for donors 1 and 2, respectively) to enrich Tfr and CD25^high^ Tfh subsets using the gating strategy shown in **Figure 1B** (see **Supplementary Table S4**). FOXP3^-^CD25^high^ Tfh cells described in previous pediatric tonsil studies were identified as a transitional population between GC Tfh and iTfr cells, exhibiting clonotypic TCRβ overlap with both. These cells expressed IL-10 and IL-21 and were capable of upregulating FOXP3 and differentiating into Tfr cells *in vitro* (35).

To resolve the transcriptional heterogeneity among tonsillar CXCR5^+^ follicular T cells and to enable identification of iTfr and nTfr cells along this continuum, we performed unsupervised clustering analysis of scRNA-seq data, which yielded 20 distinct transcriptional clusters (**Figure 2A**; **Supplementary Movie 1**). A putatively cytotoxic Tfh cell cluster 18 (52) expressing *KLRG1*/*GZMK*, and a proliferating follicular T cell cluster 12, were excluded from further analysis (**Supplementary Figure 2**).

**Figure 2:**
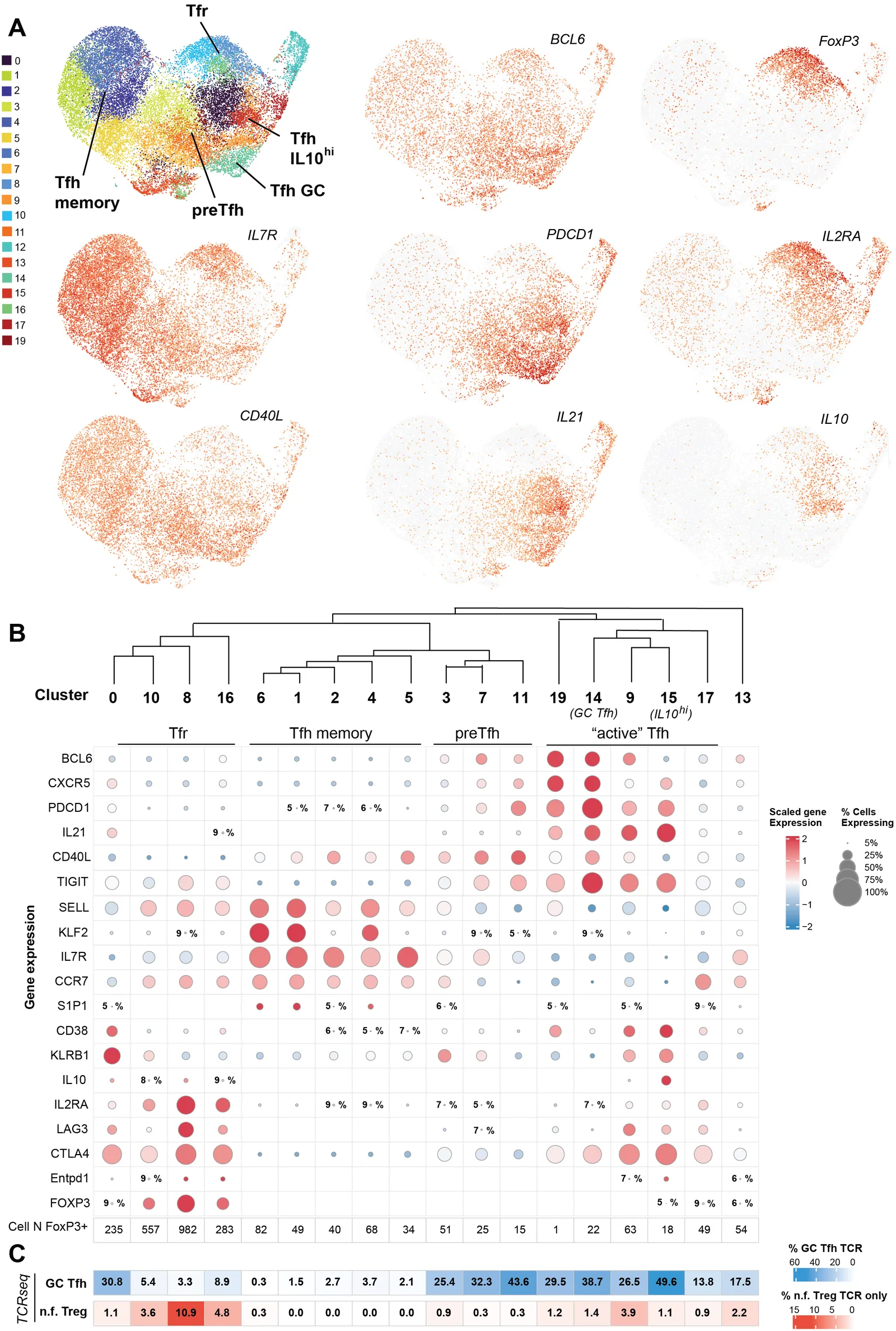
Integrated single-cell transcriptomic and TCRβ clonotype mapping of human tonsillar CD4^+^CXCR5^+^ T cell subsets. **(A)** UMAP embedding of CD4^+^CD19^−^CXCR5^+^ follicular T cells enriched for CD25^high^ cells (gating strategy shown in **Figure 1B**) from two donors with recurrent tonsillitis, profiled by 10× 5’ single-cell RNA-seq and TCR-seq. Top left: cluster identity [cluster 18 (cytotoxic Tfh) and cluster 12 (proliferating cells) were excluded from further analysis]. Annotated regions indicate the identified cell populations: Tfr, Tfh memory, pre-Tfh, GC Tfh (cluster 14), and IL10^hi^ Tfh (cluster 15). The remaining panels show log-normalized expression of selected marker genes. **(B)** Dot plot heatmap of 19 selected genes across clusters. Clusters are ordered by a hierarchical clustering dendrogram. Dot size is proportional to the percentage of cells with non-zero expression of each gene within a cluster. Dot color represents mean log-normalized gene expression, z-scored across clusters for each gene. Percentage values are shown only for clusters with >5% expressing cells. Bottom row: number of FOXP3-expressing cells per cluster. **(C)** Average percentages of TCRβ clonotypes shared between the single-cell clusters and bulk-sorted populations (tonsillar GC Tfh and n.f. Treg) for two donors. Top row (blue): percentage of each cluster’s TCRβ clonotypes shared with bulk GC Tfh. Bottom row (red): percentage of each cluster’s clonotypes shared with n.f. Tregs (excluding clonotypes also found in GC Tfh). Single-cell clusters were downsampled by random clonotype selection to the minimum clonotype count per cluster (73 and 89 for donor 1 and donor 2, respectively). GC Tfh repertoires were downsampled by top clonotype selection to match the corresponding donor’s n.f. Treg clonotypes (12,500 and 2,400 for donors 1 and 2, respectively).

Based on hierarchical clustering (53), follicular T cells stratified into several populations (**Figure 2B**; **Supplementary Figure 3**): recirculating memory Tfh cells (*IL7RA*^high^*CCR7*^high^*SELL^high^*; clusters 1, 2, 4–6); early follicular memory T cells (*IL7RA*^int^*CCR7*^int^ cluster 13, proximal to cluster 16); pre-Tfh cells (*BCL6*^+^*CXCR5*^int^*PDCD1*^int^, clusters 3, 7, 11) expressing *BCL6* and *TIGIT* (54, 55); GC Tfh cells (*BCL6*^high^*CXCR5*^high^*PDCD1*^high^, cluster 14) and associated cluster 19 with reduced *IL21*/*CD40L* (56); *IL10*-expressing Tfh cells (cluster 15) co-expressing *IL21* and immunosuppressive genes (*CTLA4*, *ENTPD1*), resembling previously described tonsillar CD25^high^ Tfh cells (26, 35, 57, 58) and closely associated with cluster 9 (**Figures 2A, B**; **Supplementary Figure 3**; **Supplementary Movie 1**). Clusters 9, 14, 15, 17, and 19 likely represent “active” Tfh cells engaged in the immune response.

We next interrogated the clonal relationships of these clusters with GC Tfh and with each other. Consistently, TCRβ repertoires of pre-Tfh, active Tfh, and early memory-like cluster 13 significantly overlapped with bulk GC Tfh cells (**Figure 2C**). scTCR-seq also revealed significant clonotypic TCRβ overlap between the pre-Tfh (3, 7, 11) and active Tfh clusters (9, 14, 15) and some overlap with clusters 19 and 17 (**Supplementary Figure 4**).

Based on *FOXP3* expression, we identified three Tfr clusters (8, 10, and 16) (**Figures 2A, B**, **3A**; **Supplementary Figure 3**). These exhibited elevated clonotypic overlap with bulk n.f. Tregs and some overlap with GC Tfh cells (**Figures 2C**, **3B**), suggesting the presence of both nTfr and iTfr cells within these transcriptional clusters (35, 59).

**Figure 3:**
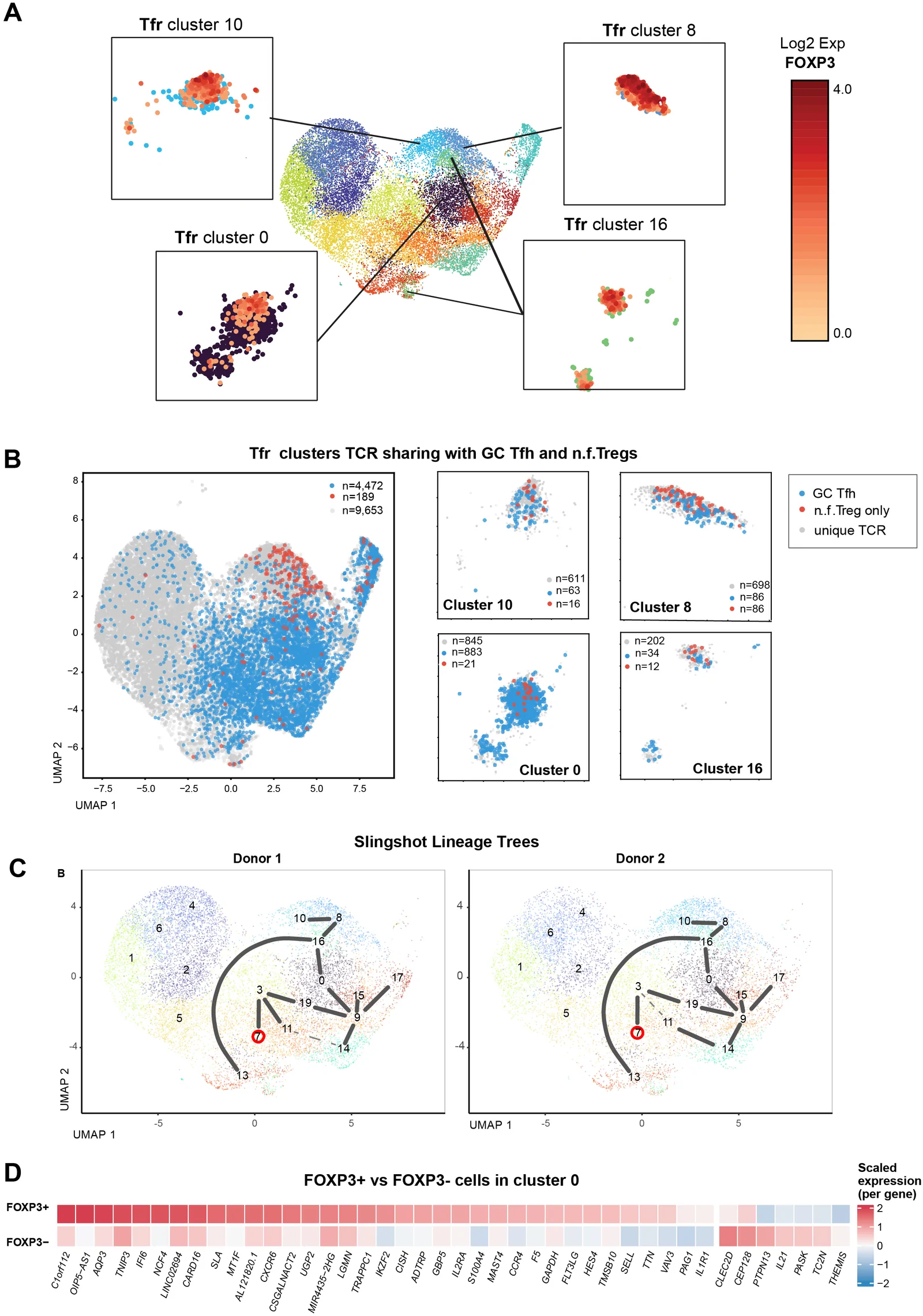
Identification of a transitional Tfh/Tfr subpopulation in human tonsils by integrated transcriptomic and TCRβ clonotype analysis. **(A)** Localization of Tfr clusters on CD4^+^ CD19^−^ CXCR5^+^ T cell UMAP. Insets show log-normalized FOXP3 expression for Tfr clusters 0, 8, 10, and 16; color scale indicates log2 expression level. **(B)** UMAP embedding of CD4^+^CD19^−^CXCR5^+^ T cells from tonsils of two donors with recurrent tonsillitis, with cells colored based on TCRβ clonotype sharing with bulk-sorted GC Tfh and n.f. Tregs (left), and zoomed views of clusters 0, 8, 10, and 16 (right). TCRβ clonotypes found in GC Tfh TCR-seq are shown in blue; those found only in n.f. Tregs TCR-seq are shown in red. Clonotype sharing was calculated per donor independently. Sharing was assessed against the complete bulk repertoires, without downsampling of either the single-cell clusters or the bulk populations. **(C)** Slingshot consensus lineage trees projected on 2D UMAP for donors 1 and 2. Edges represent the minimum spanning tree computed by Slingshot in 3D UMAP space, visualized as lines connecting cluster centroids on 2D UMAP projection. The root cluster (cluster 7, pre-Tfh) is circled in red. Solid edges indicate consensus connections present in both donors; dashed edges are present in only one donor. **(D)** Differential gene expression between *FOXP3^+^* and *FOXP3^−^* cells within cluster 0 (cells from both donors combined). Only genes significantly upregulated in *FOXP3^+^* or *FOXP3^−^* groups are shown. Differential expression was performed using Wilcoxon rank-sum test with an adjusted p-value = 0.05, and |log_2_ fold-change| 0.25 or more. *FOXP3* was excluded from the analysis as the splitting variable.

The largest follicular T cell cluster (cluster 0) hierarchically co-clustered with Tfr cells despite containing only 9% *FOXP3*^+^ cells and showing predominant clonotypic overlap with GC Tfh (**Figures 2B, C**, **3A, B**; **Supplementary Figure 3**). Cluster 0 also exhibited significant clonotypic overlap with pre-Tfh and active Tfh, plus some overlap with Tfr clusters 8, 10, 16 (**Supplementary Figure 4**). Thus, cluster 0 likely represents a transitional Tfh subpopulation differentiating into (and potentially dedifferentiating from) *FOXP3*^+^ iTfr cells (35, 60). Consistently, minimum spanning tree (MST) analysis (Slingshot (49)) places cluster 0 on the edge between active Tfh cluster 9 and Tfr cluster 16 (**Figure 3C**). Cluster 9 is positioned near GC Tfh clusters 14/19 and can branch into either Tfh cluster 15 (IL10^hi^ Tfh) or cluster 17.

Previous work suggested that iTfr cells, like CD25^hi^ Tfh cells, express IL-10 and IL-21 (35). Given that cluster 0 represents a transitional state between GC Tfh and iTfr cells, we compared the transcriptomes of *FOXP3^+^* and *FOXP3^-^* cells within this cluster (**Figure 3D**). *FOXP3* expression was associated with downregulation of *IL21* and *PTPN13* and upregulation of *IL1R1* and *IKZF2*, among others (**Figure 3D**), suggesting that *IL21* downregulation occurs as cells acquire *FOXP3* and differentiate toward the iTfr lineage.

### 3.2 CD38 expression distinguishes iTfr from nTfr cells in immunosuppressive transcriptional state, but is also elevated on activated Tfh subsets

A prior study on pediatric tonsils reported that iTfr cells differentiating from GC Tfh exhibit the highest CD38 expression (35). We reexamined this by analyzing CD38 expression at both the transcriptional (scRNA-seq) and protein surface expression levels across follicular T cell subsets in adult human tonsils. Based on the combined single-cell data from two donors with recurrent tonsillitis, elevated *CD38* expression was detected on proliferating T cells (cluster 12), the transitional cluster 0, and active Tfh subsets, with the exception of GC Tfh cluster 14 (**Figure 4A**). Flow cytometry analysis of adult human tonsils confirmed high surface expression of CD38 on FOXP3^-^CD25^+/high^ active Tfh subsets (**Figures 4B–F**). Consistent with the prior report (35), we detected both CD38^high^ and CD38^low^ cells within FOXP3+ Tfr cells (**Figures 4C, E**).

**Figure 4:**
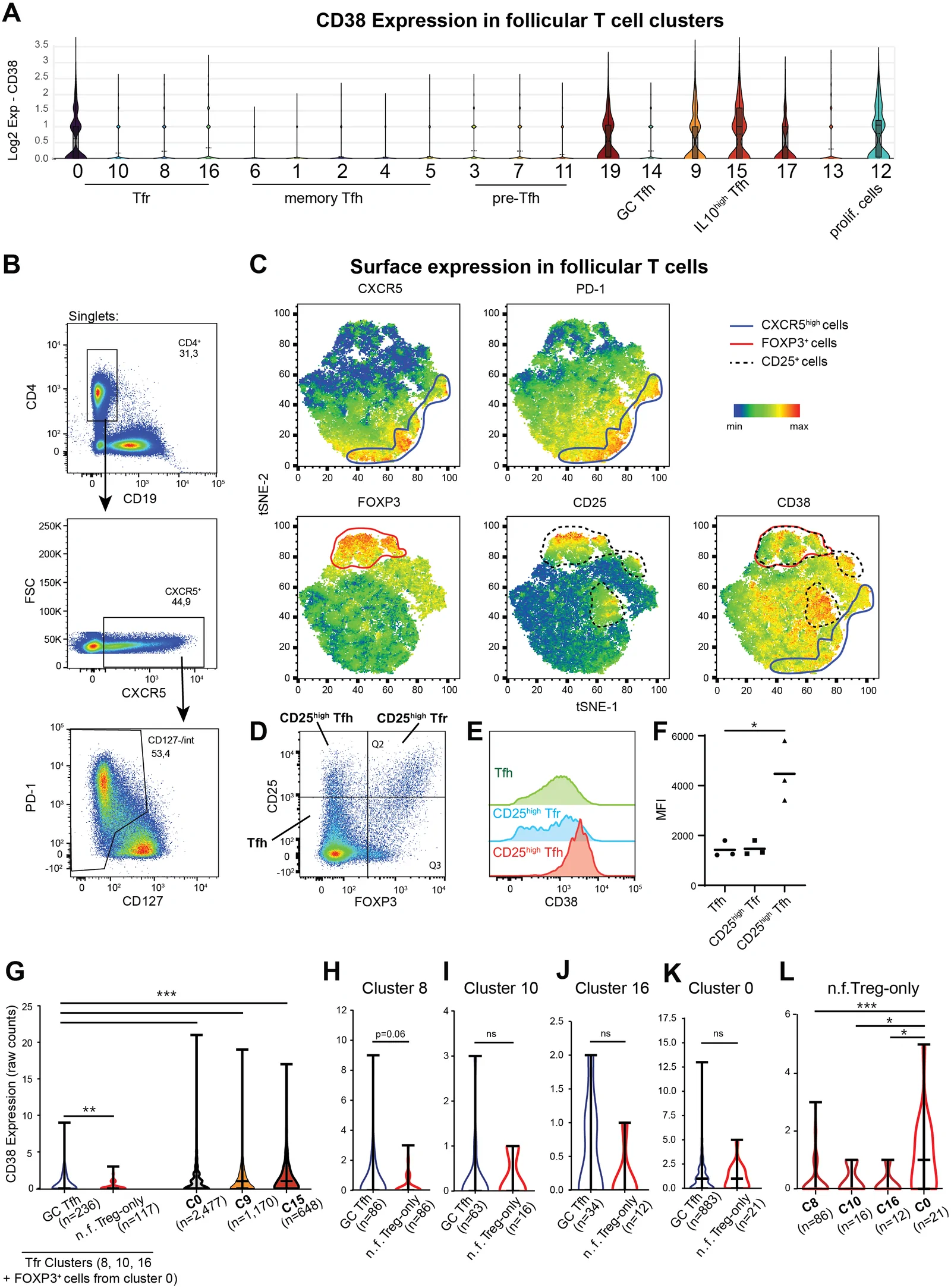
CD38 expression in follicular T cell clusters stratified by TCRβ sharing with bulk TCR-seq of GC Tfh and n.f. Treg populations. **(A)** log_2_-transformed *CD38* (RNA-seq) expression across single-cell follicular T cell clusters. **(B, C)** The gating strategy **(B)** and representative t-SNE analysis of flow cytometry data **(C)** for tonsillar CD4^+^CXCR5^+^CD127^−/int^ cells’ surface expression of CD25, CD38, and other markers from one donor. Blue contour outlines CXCR5^hi^PD1^hi^ GC Tfh cells; red contour—FOXP3^+^ Tfr cells; dashed black line—CD25^+/hi^ Tfr and Tfh cells. **(D–F)** CD38 surface expression on tonsillar CD25^high^ Tfh (CD25^high^ FOXP3^−^), CD25^high^ Tfr (CD25^high^ FOXP3^+^), and Tfh (CD25^−^ FOXP3^−^) subpopulations. **(D)** The gating strategy for identification of tonsillar CD25^high^ Tfh, CD25^high^ Tfr, and Tfh cell subsets of CD4^+^CD19^−^CXCR5^+^CD127^−/int^ cells. **(E, F)** Surface CD38 representative flow graph **(E)** and MFI **(F)**. **(G)** *CD38* expression (scRNA-seq) in cells from the s.c. Tfr pool (clusters 8, 10, 16, and FOXP3+ cells of cluster 0), categorized by TCRβ clonotype sharing with GC Tfh or with n.f. Treg only. *CD38* expression in s.c. clusters 0, 9 and 15 is shown for reference. n indicates the number of cells per category. **(H–L)** *CD38* expression (scRNA-seq) stratified by TCRβ sharing category within individual Tfr clusters: cluster 8 **(H)**, cluster 10 **(I)**, cluster 16 **(J)**, and cluster 0 **(K)**. Panel **(L)** shows CD38 expression on cells with TCRβ shared exclusively with n.f. Tregs, compared across clusters 8, 10, 16, and 0. **(A, G–L)** Data combined from tonsils of 2 donors with recurrent tonsillitis. **(G–L)** Statistical comparisons were performed using the van Elteren test with Benjamini–Hochberg adjustment. Reference groups were GC Tfh clonotype-sharing Tfr cells [in **(G)**] and n.f. Tregs clonotype-sharing cells in cluster 0 [in **(L)**]. **(B–F)** n = 3 independent experiments with tonsils of three additional donors with recurrent tonsillitis. **(F)** Statistical comparisons were performed using the two-sided Mann–Whitney U test with Benjamini–Hochberg adjustment. *p ≤0.05; **p ≤0.01; ***p ≤0.001.

To distinguish iTfr from nTfr cells within scRNA-seq clusters, we used TCRβ clonotypic overlap with GC Tfh cells and n.f. Tregs, as suggested previously (35). Tfr cells (clusters 8, 10, and 16 and *FOXP3*^+^ cells in cluster 0) were divided based on clonal sharing: cells with clonotypes shared with GC Tfh were assigned as iTfr, whereas those overlapping exclusively with n.f. Tregs (and not with GC Tfh) were assigned as nTfr.

Consistent with previously reported upregulation of CD38 on iTfr compared to nTfr (35), Tfr cells overlapping with bulk GC Tfh TCRβ displayed higher *CD38* expression than those overlapping exclusively with n.f. Tregs (**Figure 4G**). However, CD38 expression on the transitional Tf cluster 0 and active Tfh clusters 9 and 15 was higher than that on iTfr cells (**Figure 4G**).

Given that iTfr cells are enriched in cluster 0, which itself exhibits elevated *CD38*, we examined whether high CD38 expression was confined to iTfr cells within this cluster. To address this, we compared CD38 expression across individual Tfr clusters. Within the most abundant Tfr cluster with the most immunosuppressive gene signature (cluster 8), iTfr showed a trend for higher *CD38* expression than nTfr (**Figure 4H**, p = 0.06). By contrast, this difference was less pronounced in clusters 10 and 16 (**Figures 4I, J**).

Critically, cluster 0 contained many iTfr cells and a few Tfr cells that overlapped exclusively with n.f. Treg (**Figure 3B**). However, among these rare events, *CD38* expression on nTfr was not significantly different from that on iTfr cells (**Figure 4K**). While uneven cell number distribution may limit the statistical power of the iTfr versus nTfr comparison in cluster 0, CD38 expression was clearly elevated on nTfr cells from cluster 0 compared to nTfr from other clusters (**Figure 4L**).

To validate these findings in an independent donor cohort, we analyzed single-cell RNA/TCR data from four adult donors (34–58 years old, including one donor with psoriasis and one with rheumatoid arthritis) from the recently published TABLO atlas of human tonsil T cells (50) (**Supplementary Figure 5**). Among tonsillar *CD4^+^* T cells, the study identified three subpopulations of Tregs, annotated as naïve, *CD161^+^*, and memory Tregs, as well as seven subpopulations of Tfh and GC Tfh cells. Comparison of the three Treg subpopulations revealed little to no expression of *CXCR5* and *PDCD1* in naïve Tregs, with elevated expression of these genes in the other two Treg subsets (**Supplementary Figures 5A, B**). CXCR5-expressing tonsillar Tregs (*CD161^+^* and memory Tregs), Tfh, and GC Tfh cells from these donors (344k cells) were projected onto our 20-cluster follicular T cell reference (**Supplementary Figures 5C–E**). The TABLO Treg subsets fell predominantly within Tfr clusters 8 and 10 and the transitional cluster 0, with some also falling into cluster 9. Few Tfr cells were distributed to cluster 16. TABLO-annotated GC Tfh cells fell predominantly within GC Tfh cluster 14, while other TABLO Tfh cells distributed predominantly between other activated Tfh and pre-Tfh cell clusters (**Supplementary Figures 5C, D**). Within the projected TABLO data, restricted to the Tfr pool, we then applied the same clonal criteria as above, but using paired TCRα/TCRβ clones rather than TCRβ clonotypes to identify iTfr and nTfr. As a GC Tfh reference TCR repertoire, we used the TABLO donor cells projected onto our reference GC Tfh cluster 14. As a non-follicular Treg reference repertoire, we used cells annotated as naïve Tregs in the TABLO atlas that had no detectable *CXCR5* (30,471 of 33,244 cells, 91.7%; **Supplementary Figures 5A,B**). Cells of the Tfr pool (assigned to reference Tfr clusters 8, 10, 16, and *FOXP3+* cells of cluster 0) were categorized by sharing of their TCRs with GC Tfh or with n.f. Tregs only, as in our own donors. In the atlas cohort, this yielded 228 GC Tfh-sharing and 2,134 n.f. Treg-only-sharing Tfr cells across the four donors.

Importantly, in all four TABLO donors, including those with autoimmune diseases, iTfr cells collectively expressed higher *CD38* than nTfr cells and had comparable or lower CD38 expression than active Tfh cells in clusters 9, 15, and 0 (**Supplementary Figures 5F, K, P, U**). Moreover, elevated *CD38* expression on GC Tfh clone-sharing cells versus n.f. Treg clone-sharing cells was detected within Tfr cluster 8 (**Supplementary Figures 5G, L, Q, V**) but not within cluster 0 (**Supplementary Figures 5I, N, S, X**), and n.f. Treg clone-sharing T cells within cluster 0 had higher CD38 expression than those within clusters 8 and 10 (**Supplementary Figures 5J, O, T, Y**).

In summary, the CD38 expression patterns observed in our index donors were recapitulated across tonsil samples from diverse clinical conditions, including non-infectious tonsillectomy, tonsillar hypertrophy, recurrent tonsillitis, and autoimmune disease. Across all donors, the majority of iTfr cells, particularly those within cluster 8, exhibited elevated CD38 expression compared to nTfr cells. Importantly, a small population of n.f. Treg-TCR-sharing cells within the transitional cluster 0 also expressed high levels of CD38 in all tonsils examined, suggesting that CD38 upregulation is not strictly confined to the iTfr lineage within this cluster (**Figures 4G–L**; **Supplementary Figures 5F–Y**).

### 3.3 Analysis of CD38, KI67, and CD45RA on the circulating follicular T cell and Treg subsets

CD38^+^ Tregs and Tfrs have been previously reported in human blood (34, 61), with CD38^+^ subsets expressing the proliferation marker KI67 (61). Conversely, a substantial fraction of blood CD4^+^CXCR5^+^FOXP3+ cells express CD45RA, likely representing immature pre-Tfr cells (34), though CD38 and KI67 expression on this subset has not been characterized by flow cytometry.

To assess the relationship between surface expression of CD38, CD45RA, and the proliferation factor KI67 in circulating Tfr cells, we collected peripheral blood from healthy adult donors. Follicular T cell subsets were stratified based on CD25 expression into CD25^−^ cTfh, CD25^int^ cTfh, and CD25^high^CD127^low^ cTfr (**Figure 5A**), representing approximately 40%, 45%, and 1%–5% of circulating follicular T cells, respectively (**Figure 5B**).

**Figure 5:**
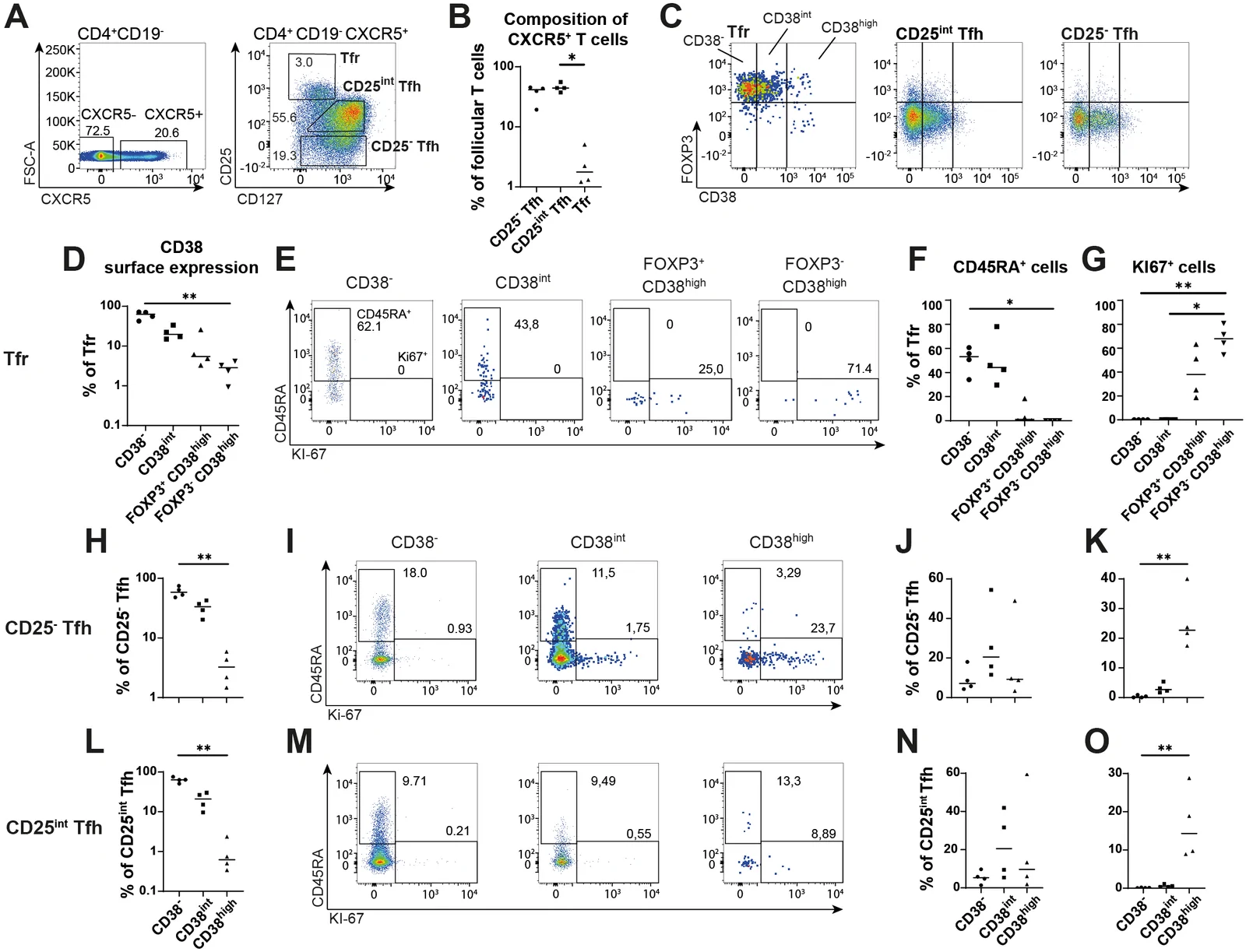
Expression of CD38, CD45RA, and KI67 in Tfh and Tfr cell populations in peripheral blood. **(A, B)** Peripheral blood CD25^−^ Tfh, CD25^int^ Tfh, and Tfr cells gating strategy **(A)** and percentage of CD4^+^ follicular T cells **(B)**. **(C)** CD38 and FOXP3 expression in CD25^−^ Tfh, CD25^int^ Tfh, and Tfr subsets. **(D–O)** Analysis of intracellular KI67 and surface CD45RA expression on CD38^−^, CD38^int^, and CD38^hi^ subsets of Tfr **(D–G)**, CD25^−^ Tfh **(H–K)**, and CD25^int^ Tfh **(L–O)**. CD38 subsets’ frequencies **(D, H, L)**, CD45RA vs. KI67 gating strategy **(E, I, M)**, and percentages of CD45RA^+^ **(F, J, N)** and KI67^+^ **(G, K, O)** cells. Analysis was performed on PBMCs from four healthy donors. Lines represent the median values. The Kruskal–Wallis test with Dunn’s correction for multiple comparisons was used for statistical analysis of follicular T cell composition **(B, D, F–H, J–L, N, O)**. *p ≤0.05; **p ≤0.01.

Analysis of CD38^−^, CD38^int^, and CD38^high^ cTfr cell subsets revealed that a substantial proportion of cells within the CD38^high^ subpopulation were FOXP3^−^ (**Figures 5C, D**). Importantly, stringent gating on CD25^high^ cTfr was critical for enriching FOXP3^+^ cells within the CD38^high^ subset (**Supplementary Figures 6A, B**). This enrichment was reflected in our FOXP3 frequency data: while >75% of CD25^high^CD127^low^CD38^−/int/hi^ cells were FOXP3^+^, fewer than 4% of CD25^-^ and CD25^int^ cells were FOXP3^+^ (**Supplementary Figure 6C**), confirming that CD25^-^ and CD25^int^ populations are predominantly cTfh, whereas CD25^high^ cells are enriched for cTfr.

When we assessed KI67 and CD45RA separately on FOXP3^+^ and FOXP3^-^ cells within the CD38^high^ subset (**Figure 5E**), the CD45RA^+^ pre-Tfr fraction was found exclusively within CD38^−^ and CD38^int^ subsets (both KI67^-^), comprising approximately 50% of these populations. Within CD38^high^ cTfr, KI67 was detected in 30% of FOXP3^+^ cells and 70% of FOXP3^-^ cells (**Figures 5E–G**).

In the CD25^−^ and CD25^int^ cTfh populations, only the CD38^high^ subset contained KI67^+^ cells (~25% and ~15%, respectively). However, unlike cTfr, substantially fewer cTfh cells were CD45RA^+^, with peak frequency (~20%) observed in the CD38^int^ subset (**Figures 5H–O**).

High CD38 expression was not inherent to all KI67^+^ T cells, as approximately 15% of CD38^−^ blood Tregs were also KI67+ (**Supplementary Figures 6D–H**). Across the Treg compartment, most (~80%) blood n.f. Tregs were CD38^−^ (**Supplementary Figure 6E**), and only ~15% of CD38^int^ Tregs co-expressed CD45RA (**Supplementary Figure 6H**).

Having confirmed that CD38^high^ Tfr cells lack CD45RA^+^ pre-Tfr cells, we next assessed whether CD38 expression distinguishes circulating iTfr cells.

### 3.4 Analysis of clonal origin of circulating Tfr and Tfh cells

Peripheral blood CD4^+^CD19^−^CXCR5^+^ follicular T cells from two donors with recurrent tonsillities (pre-tonsillectomy, see **Figure 1A**) were sorted into four subsets for bulk TCRβ-seq: CD25^−^, CD25^int^, CD25^high^CD127^−^CD38^−^, and CD25^high^CD127^−^CD38^high^, in multiple replicates using the gating strategy shown in **Figure 6A**.

**Figure 6:**
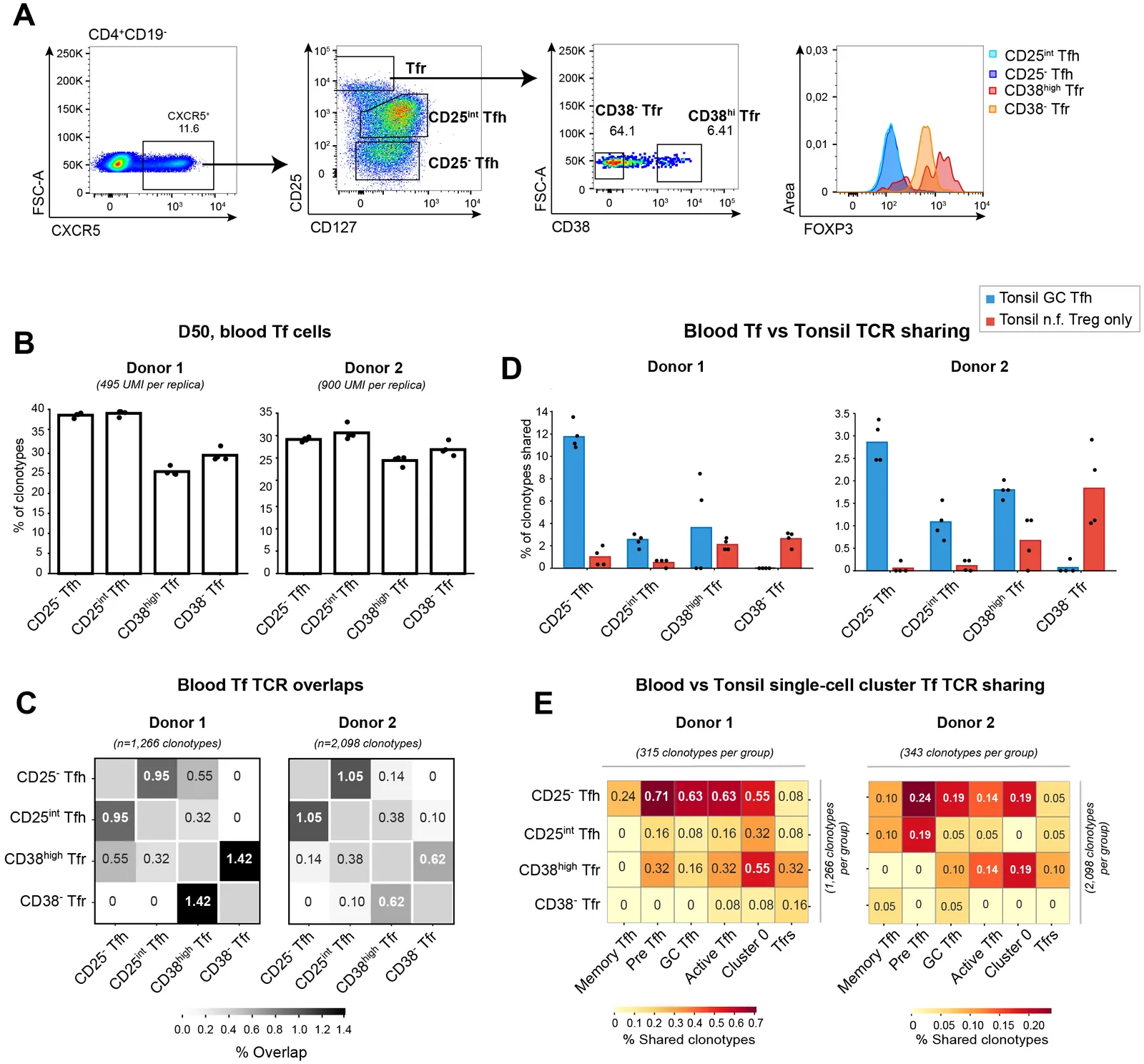
TCRβ repertoire analysis of peripheral blood follicular T cell subpopulations. The analysis was performed on four CD4^+^ CXCR5^+^ T cell subsets from donors 1 and 2: CD25^−^ Tfh (CD25^−^), CD25^int^ Tfh (CD25^int^), CD38^high^ Tfr (CD25^high^ CD127^−^ CD38^high^), and CD38^−^ Tfr (CD25^high^ CD127^−^ CD38^−^). **(A)** Flow cytometry gating strategy for sorting circulating blood follicular T cell subpopulations: CD25^−^ Tfh, CD25^int^ Tfh, CD38^high^ Tfr, and CD38^−^ Tfr (left panels) and representative FOXP3 expression overlay histogram (right panel) for selected follicular T cell subpopulations from peripheral blood. **(B)** D50 diversity index. Replicates were downsampled to 495 (donor 1) or 900 (donor 2) UMI by random selection. Dots represent individual replicates. Bars show mean values. **(C)** Pairwise TCRβ clonotypic overlap (percentage) between blood follicular T cell subpopulations. All blood subpopulations were downsampled to clonotype values indicated on the figure. **(D)** Percentage of blood follicular T cell subset clonotypes shared with tonsillar populations: GC Tfh (blue bars) or n.f. Treg only (red bars). GC Tfh repertoires were downsampled to 12,500 (donor 1) and 2,400 (donor 2) clonotypes to match the corresponding donor’s n.f. Treg clonotype count. Dots represent individual replicates. Bars show mean values. **(E)** Percentage of blood follicular T cell subset TCRβ clonotypes shared with single-cell cluster groups. Single-cell cluster groups were defined as: memory Tfh (clusters 1, 2, 4, 5, 6), pre-Tfh (clusters 3, 7, 11), GC Tfh (cluster 14), cluster 0, other “active” Tfh (clusters 9, 15, 19), and Tfrs (clusters 8, 10, 16). TCRβ clonotypes from blood and s.c. T cell groups were independently downsampled by numbers indicated on the figure.

Intracellular FOXP3 analysis revealed that the frequency of FOXP3^+^ cTfr cells in donors with recurrent tonsillitis was comparable to that of healthy donors (**Supplementary Figure 6C**).

TCRβ repertoire analysis showed that CD25^−^ cTfh replicates exhibited greater clonotypic overlap than CD25^int^ cTfh replicates (**Supplementary Figure 7**), whereas D50 diversity measurements showed no substantial differences between these subsets (**Figure 6B**). In contrast, both CD38^high^ and CD38^−^ cTfr displayed higher clonality than both cTfh subsets, with CD38^high^ cTfr being the most clonal (least diverse) (**Figure 6B**).

Substantial clonotypic overlap was detected between the CD25^−^ and CD25^int^ cTfh subsets, as well as between CD38^high^ and CD38^−^ cTfr (**Figure 6C**). In both donors, some overlap was also observed between cTfh (both CD25^−^ and CD25^int^) and CD38^high^ cTfr, whereas CD38^−^ cTfr showed negligible overlap with cTfh (**Figure 6C**). Thus, CD38^high^ cTfr, but not CD38^−^ cTfr, has TCRβ repertoire overlap with peripheral cTfh.

To examine relationships between circulating and tonsillar subsets, we assessed clonotypic TCRβ overlap among circulating subsets (CD25^−^ cTfh, CD25^int^ cTfh, CD38^high^ cTfr, and CD38^−^ cTfr) with tonsillar GC Tfh and n.f. Tregs. CD25^−^ cTfh exhibited the highest overlap with tonsillar GC Tfh, with 2.5- to 4-fold higher frequency than CD25^+^ cTfh (**Figure 6D**). Notably, CD38^high^ cTfr showed comparable or greater overlap with tonsillar GC Tfh than CD25^+^ cTfh, while CD38^−^ cTfr showed minimal or no overlap (**Figure 6D**), supporting a GC Tfh origin for the CD38^high^ cTfr.

However, clonotypic overlap with n.f. Tregs showed a different pattern: CD38^−^ cTfr had the highest overlap with n.f. Tregs, while CD38^high^ cTfr also showed substantial overlap, exceeding that of cTfh (**Figure 6D**). This finding, together with CD38^high^ cTfr overlapping with CD38^−^ cTfr, suggests a mixed origin for CD38^high^ cTfr, comprising contributions from both GC Tfh- and Treg-lineage cells.

Assessing clonotypic overlap with tonsillar single-cell populations, CD25^−^ cTfh substantially overlapped with activated Tfh, GC Tfh, and transitional cluster 0, and especially pre-Tfh (**Figure 6E**). Notably, CD38^high^ cTfr showed substantial overlap with transitional cluster 0, exceeding its overlap with other Tfh or Tfr cluster groups. Conversely, CD38^−^ cTfr overlapped with n.f. Treg (not shared with GC Tfh) but showed minimal overlap with single-cell Tfr clusters, suggesting no enrichment of tonsillar Tfrs in this circulating population (**Figures 6D, E**).

Collectively, our data suggest that circulating CD38^−^ Tfr cells (which constitute more than 65% of Tfr cells, with equal proportions of pre-Tfr and cTfr) are likely of natural Treg origin (nTfr) and are not enriched for tonsillar nTfr. In contrast, the proliferating CD38^high^ subpopulation, which constitutes 10% or less of Tfr cells, likely represents a mixture of iTfr and nTfr, with a notable portion of iTfr cells shared with tonsillar Tfh and the transitional Tfh/iTfr subpopulations.

## 4 Discussion

Understanding the clonal and transcriptional relationships between tonsillar Tfh, Tfr, and Treg populations and their circulating counterparts is essential for interpreting studies that rely on blood sampling. Here, we combined TCRβ clonotype analysis and single-cell transcriptomics to map the differentiation trajectories of follicular T cell subsets and trace their clonal connections between tonsil and peripheral blood.

scRNA-seq of total tonsillar CD4^+^CD19^−^CXCR5^+^ follicular T cells identified recirculating memory Tfh, pre-Tfh, and classical GC Tfh clusters (**Figures 2A, B**; **Supplementary Figure 3**), consistent with a prior report (52). Enrichment for CD4^+^CD19^−^CXCR5^+^CD25^high^ cells additionally resolved multiple Tfr and CD25-expressing Tfh subsets (**Figures 2A, B**, **3A**; **Supplementary Figure 3**). We identified three Tfr subsets: an activated effector subset (cluster 8) with high expression of suppressive factors *BATF*, *IRF4*, *LAG3*, *FOXP3*, *IL2RA*, *TNFRSF18*, *TNFRSF4*, *LINC01943*, *IL1R2*, and *CXCR6*; a less activated cluster 10; and cluster 16 (**Figures 2A**, **3A**; **Supplementary Figure 3**). Tfr clusters showed elevated clonotypic overlap with n.f. Tregs (especially cluster 8) but also shared clonotypes with GC Tfh (**Figures 2C**, **3B**). Collectively, these findings suggest that nTfr and iTfr cells do not segregate into distinct transcriptional clusters; rather, they distribute across shared activation states, with their relative proportions differing by cluster.

Hierarchical clustering assigned cluster 0 to Tfr cells despite containing fewer than 10% FOXP3^+^ cells. This cluster predominantly shares clonotypes with GC Tfh and other activated Tfh populations (**Figures 2**, **3A, B**; **Supplementary Figure 4**), although a few cells shared clonotypes exclusively with n.f. Tregs (**Figure 3B**). Cluster 0, defined by high expression of *TSHR*, *CDKL2*, *CD38*, *AC022239.1*, and the highest *KLRB1*, likely represents a transitional state between activated Tfh and iTfr. This assignment is supported by its localization on the UMAP embedding between activated *IL2RA^+^* Tfh clusters (9, 15, 17) and Tfr cluster 16 (**Figure 2A**; **Supplementary Movie 1**), and is further corroborated by MST connectivity directly linking cluster 9 to cluster 16 (**Figure 3C**).

We identified an active Tfh cluster 15 with high expression of *IL10*, *IL21*, *PRDM1*, *TSHR*, *CDKL2*, AC022239.1, and *CD38*, aligning with prior descriptions of CD25^high^ Tfh cells (35, 58). MST analysis suggests that IL10^hi^ Tfh cells (cluster 15) may differentiate from cluster 9, which itself represents an intermediate state linking GC Tfh to the transitional cluster 0.

However, IL10^hi^ Tfh (cluster 15) is only one of several *IL2RA*-expressing active Tfh/transitional clusters (clusters 17, 9, and 0) (**Figure 2B**; **Supplementary Figure 3**). Thus, flow sorting based solely on CD25^high^ expression likely captures a heterogeneous mix of subsets with distinct immunosuppressive and immunostimulatory properties, potentially confounding *ex vivo* functional analyses (26, 35, 58). Moreover, the inclusion of both FOXP3^-^ and FOXP3^+^ cells from cluster 0 may explain previous reports that iTfr cells produce IL-21 (35). However, direct comparison of *FOXP3^+^* versus *FOXP3^−^* cells within cluster 0 indicates that *IL-21* expression is suppressed in newly formed iTfr (**Figure 3D**). Together, these findings indicate that more precise separation of CD25-expressing Tfh and Tfr subsets using additional markers is needed to accurately evaluate their respective functions.

Distinguishing iTfr from nTfr cells using gene or surface markers is essential for identifying disease-relevant circulating Tfr subsets. A prior study of sorted pediatric tonsillar Tfr cells (CD25^high^CXCR5^int^PD1^int^) reported elevated *TNFRSF18*, *TIGIT*, *TNFRSF4*, *IL7R* on nTfr versus iTfr, and higher *KLRB1*, *LAG3,* and *CD38* on iTfr versus nTfr (35). These findings align with the differences we observe in adult tonsils between nTfr-enriched cluster 8 and the transitional cluster 0, which is enriched for iTfr cells (**Figure 3B**; **Supplementary Figure 3**), though both iTfr and nTfr cells are also distributed across other transcriptional states (**Figure 3B**).

In this study, we evaluated CD38 as both a transcriptional and surface protein marker for identifying iTfr cells in adult human tonsils. Consistent with a previous report in pediatric tonsils (35), we observed that overall, total iTfr cells expressed higher *CD38* than their nTfr counterparts (**Figure 4G**; **Supplementary Figures 5F, K, P, U**). The same relationship was seen within Tfr cluster 8, which has the most immunosuppressive gene signature (**Figure 4H**; **Supplementary Figures 5G, L, Q, V**) supporting the utility of CD38 as a discriminating marker for most tonsillar Tfr. However, CD38 expression was not confined to iTfr cells; it was also elevated on active Tfh and transitional Tfh populations (**Figure 4G**; **Supplementary Figures 5F, K, P, U**), suggesting that CD38 upregulation is associated with T cell activation or recent exit from the GC reaction rather than being a strict lineage-defining feature of iTfr cells.

A notable caveat emerged from our analysis of the transitional cluster 0. Within this population, nTfr-like cells (defined by clonotypic overlap exclusively with n.f. Tregs) did not exhibit lower CD38 expression than iTfr cells. Although these nTfr-like cells were rare within cluster 0, limiting statistical power, this trend was consistently observed across multiple donors (**Figure 4K**, **Supplementary Figures 5I, N, X, X**). Furthermore, CD38 expression on these cluster 0 nTfr-like cells was elevated compared to nTfr cells in other Tfr clusters, indicating that the transitional microenvironment may promote CD38 upregulation independently of Tfr origin (**Figure 4L**; **Supplementary Figures 5J, O, T, Y**). Collectively, these findings suggest that while CD38 is a useful marker for distinguishing iTfr from nTfr in most tonsillar Tfr subsets, its specificity is context-dependent. In transitional populations, where cells are actively modulating their phenotype, CD38 expression may reflect activation state or differentiation status rather than lineage origin, and should therefore be interpreted with caution when used as a standalone marker.

Human peripheral blood has been extensively used to analyze cTfh and cTfr cells in the context of vaccinations and disease (30–34). However, distinguishing nTfr from iTfr cells in the circulation has remained challenging. Previous studies have identified CD38^+^ Treg cells in the blood (34) and reported increased frequencies of CD38^+^ cTfr in anti-PD-1-treated cancer patients (61). Moreover, *in vitro* stimulation of CD38^+^ cTfr cells was reported to enhance their IL-21 production and promote plasma cell formation in co-cultures with B cells and Tfh cells, compared to their CD38^-^ counterparts (61). Nevertheless, two key questions remained unresolved: first, whether surface CD38 identifies cTfr cells derived from GC Tfh in human blood; and second, whether CD38^-^ cTfr cells constitute a pure nTfr population or a heterogeneous mixture.

Comparative TCRβ repertoire analysis of blood CD38^high^ Tfr and CD38^−^ Tfr (CD4^+^CXCR5^+^CD25^high^CD127^low^) with matched tonsillar GC Tfh, n.f. Tregs, and other follicular T cell subsets revealed that CD38^−^ cTfr (half of which are CD45RA^+^ pre-Tfr cells (34); **Figures 5D–F**) has no clonotypic overlap with GC Tfh (**Figure 6D**). This subpopulation shared TCRβ clonotypes exclusively with n.f. Tregs (**Figure 6D**), suggesting that most CD38**^-^** Tfr cells (both pre-Tfr and CD45RA^−^ cTfr) are derived from natural Tregs, although some pTreg contribution cannot be ruled out.

In contrast, CD38^high^ Tfr (which are largely CD45RA**^−^** cells; **Figures 5D–F**) exhibited clonotypic overlap with circulating Tfh cells (**Figure 6C**) and tonsillar GC Tfh (**Figure 6D**). Notably, their highest clonotypic overlap was with tonsillar single-cell cluster 0 (the transitional Tfh/iTfr with high *CD38* expression) (**Figure 6E**). Based on these findings, some CD25^+^CD38^high^ transitional Tfr cells likely exit into the bloodstream. However, this subpopulation represents only a small fraction of total circulating Tfr, and 20–60% of these cells are KI67^+^ (proliferating) (**Figures 5D, E ,G**), suggesting transient presence in the circulation. Approximately 25% of CXCR5^+^CD25^high^CD127^low^CD38^high^ cells are FOXP3**^−^** (**Figures 5C, D**), possibly representing FOXP3^-^ cells originating from the transitional cluster 0.

CD38^high^ cTfr showed clonotypic overlap with tonsillar GC Tfh comparable to or greater than that of CD25^+^ cTfh (**Figure 6D**), although CD38^high^ cTfr was more clonal (**Figure 6B**). At the same time, CD25^int^ cTfh had minimal overlap with n.f. Tregs, whereas CD38^high^ Tfr showed 2- to 3-fold higher overlap with n.f. Tregs (**Figure 6D**) and also shared clonotypes with CD38^−^ Tfr cells (**Figure 6C**). Collectively, these results indicate that the CD38^high^ cTfr compartment contains both iTfr and nTfr cells. While this observation parallels our findings in transitional single-cell cluster 0 from tonsils (**Figure 3B**), the circulating CD38^high^ cTfr subset appears to contain a greater proportion of nTfr cells.

Finally, CD25^−^ cTfh cells were less clonal than CD38^high^ cTfr cells (**Figure 6B**) and had 2- to 3-fold higher overlap with tonsillar GC Tfh than either CD25^int^ cTfh or CD38^high^ cTfr (**Figure 6D**). Although CD25^−^ cTfh overlapped with CD25^int^ cTfh (**Figure 6C**), single-cell comparison revealed that their highest overlap was with pre-Tfh subsets (**Figure 6E**), suggesting that many CD25^−^ cTfh cells arise from early activated/pre-Tfh clones.

In summary, our analysis reveals that in adult human tonsils, nTfr and iTfr cells do not segregate into distinct transcriptional clusters but rather distribute across shared activation states at varying proportions. Although some activated Tfh subsets, including CD25^hi^ Tfh cells, express high levels of CD38, CD38 expression nonetheless distinguishes most iTfr from nTfr cells, consistent with a prior report identifying CD38 as a positive marker of iTfr cells (35). However, the specificity of CD38 is also not absolute, particularly within the transitional cluster 0, where nTfr-like cells (defined by clonotypic overlap exclusively with n.f. Tregs) also upregulate CD38. Consistent with this, in the circulation, CD38^−^ cTfr cells show clonotypic overlap exclusively with tonsillar n.f. Tregs and lack detectable sharing with GC Tfh, supporting a predominantly natural Treg origin. By contrast, CD38^high^ cTfr cells exhibit clonotypic overlap with both tonsillar GC Tfh and n.f. Tregs, as well as with CD38^−^ cTfr, indicating that this subset is of mixed origin.

A few limitations should be considered when interpreting these findings. Clonotypic overlap provides a valuable first approximation and is particularly informative for establishing the absence of clonal sharing between populations. However, the presence of shared TCRβ clonotypes does not definitively confirm clonal identity, as the paired TCRα chain may differ. Additionally, while our data indicate that CD38^−^ cTfr cells are predominantly of nTfr origin, we cannot formally exclude the possibility that some cells clonotypically overlapping with n.f. Tregs derive from peripherally induced Tregs rather than thymic-derived natural Tregs. Possible plasticity of some Tfr cells toward a Tfh-like phenotype (exTfrs) adds an additional layer of complexity to the interpretation of clonal relationships (60).

The analysis of circulating subsets is also constrained by practical and biological limitations. First, the low frequency of Tfr cells in peripheral blood limits clonotype availability and precludes complete exclusion of low-frequency overlap between CD38^−^ cTfr and GC Tfh. Second, our comparison of circulating and tonsillar Tfr repertoires was performed in only two donors, both of whom underwent tonsillectomy for recurrent tonsillitis rather than for non-immune indications. While tonsils provide a relevant and informative site for studying GC-derived Tfh and Tfr cells, chronic inflammation associated with recurrent tonsillitis may plausibly affect the magnitude of GC responses and the Treg: Tfr ratio, potentially influencing the composition of the circulating Tfr pool. Thus, our findings may not fully recapitulate the clonal relationships present under steady-state conditions or in other inflammatory contexts.

Furthermore, while tonsils serve as a representative secondary lymphoid organ for studying GC-derived Tfh and Tfr cells, we cannot rule out that Tfr populations originating from other lymphoid sites, such as lymph nodes or gut-associated lymphoid tissue, may contribute differently to the circulating pool. This is particularly relevant given the limited clonotypic overlap we observed between tonsillar nTfr and circulating CD38^−^ cTfr, which may reflect either genuine biological compartmentalization or the incomplete representation of tonsil-derived clones in the circulation. Future studies incorporating additional donors, diverse clinical conditions, and multiple lymphoid tissue sites will be important to further delineate the origins and dynamics of circulating Tfr cells.

Despite these limitations, our findings establish a TCR clonotypic framework for interpreting circulating Tfr subsets and highlight the value of integrating CD38 phenotyping with TCRβ repertoire analysis. We propose that blood-based studies of human autoimmunity, vaccination, or cancer immunotherapy would benefit from incorporating both CD38 surface marking and clonal overlap with tissue-derived reference sets (relevant to the site of vaccination or disease) to more accurately interpret Tfr origin and response.

## Data Availability

The datasets presented in this study can be found in online repositories. All raw sequencing data (bulk TCRβ and single-cell RNA with paired T-cell receptor sequencing) generated in this study have been deposited in the NCBI under BioProject accession PRJNA1459622, comprising BioSamples SAMN57560266 (D1 tonsil), SAMN57560267 (D1 blood), 844 SAMN57560268 (D2 tonsil), and SAMN57560269 (D2 blood).
