# Supplementary material for: TCR clonotyping of paired blood and tonsillar T-follicular cells reveals the mixed origin of CD38high Tfrs
Source: Front Immunol. 2026 Oct 9;17:1947623. doi: 10.3389/fimmu.2026.1947623 (PMC13690733; doi:10.3389/fimmu.2026.1947623)
Supplement: Supplementary file 1 [file DataSheet2.pdf]

Supplementary Material

TCR clonotyping of paired blood and tonsillar T-follicular cells reveals the mixed origin of CD38^{high} Tfrs

Sophiya R. Sokolova *et al.*

*Corresponding author: Irina L. Grigorova
Email: grig76@gmail.com

1 Supplementary Figures

1.1 Supplementary Figures 1-7

2 Other Supplementary Data

2.1 Legend to Supplementary Movie 1

2.2 Legends to Supplementary Tables (Tabs S1-S4)

2.3 Legend to Supplementary Data

1.1 Supplementary Figures

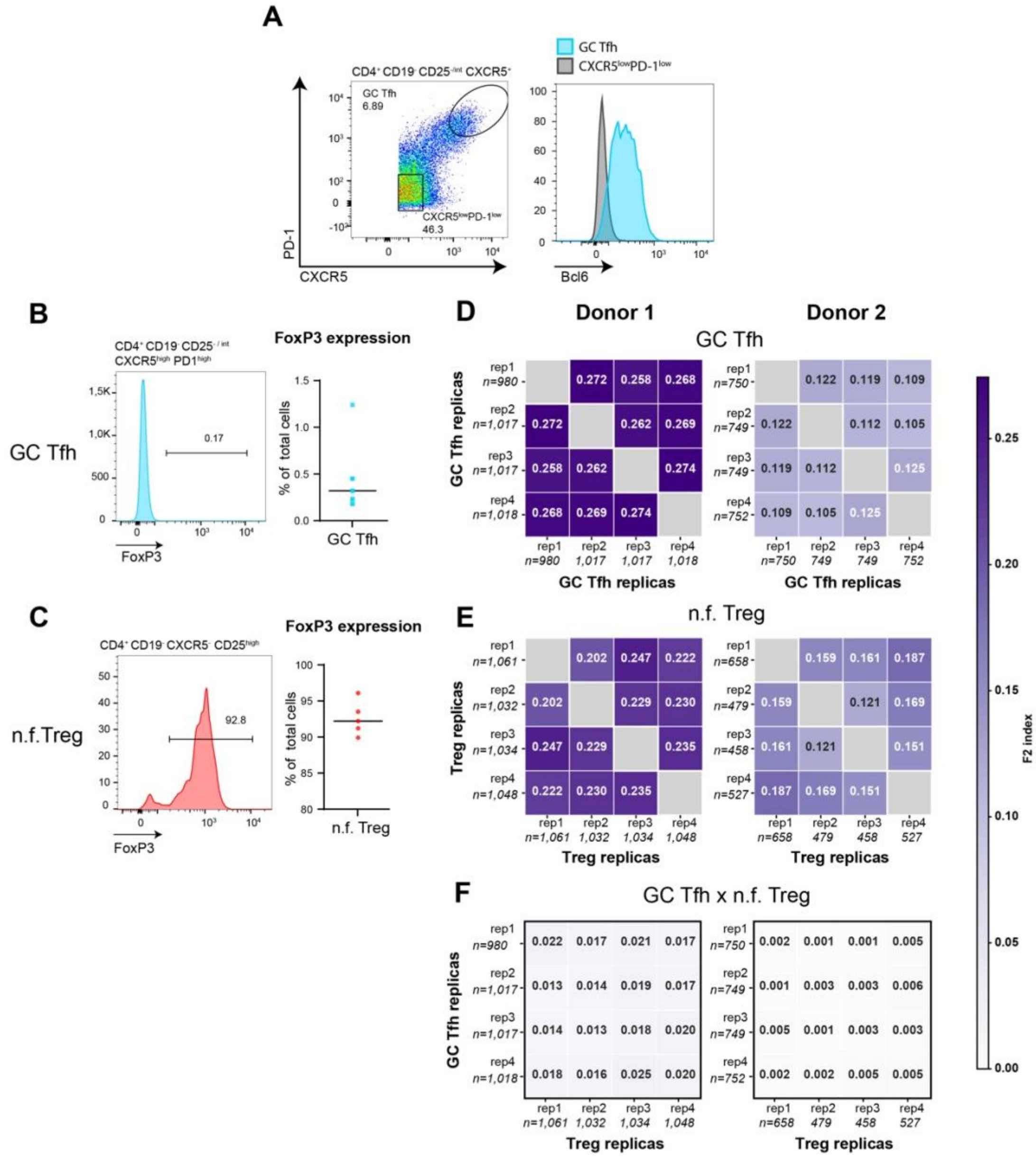

Supplementary Figure 1. Phenotypic validation of tonsillar GC Tfh and n.f. Tregs for TCRβ sequencing. (**A**) Verification of BCL6 transcription factor expression in selected tonsillar GC Tfh cells. (**B, C**) Representative intracellular FOXP3 staining histograms and percentage of FOXP3-expressing cells in selected GC Tfh (**B**) and n.f. Treg (**C**) cells (n=5 human tonsils). (**D–F**) Pairwise TCRβ repertoire overlap between technical replicas of tonsillar GC Tfh (**D**) and n.f. Treg (**E**), and between GC Tfh and n.f. Treg replicas (**F**), for donors 1 and 2 (4 replicas per population per donor). Data from (A–C) are from independent experiments from 5 additional donors` tonsils. Overlap is shown as the UMI-weighted F2 index (see Methods), where 0 indicates no shared clonotypes and 1

indicates identical repertoires. Within each donor, all replicas were downsampled by random UMI selection to a common depth (1,279 UMI for donor 1, 801 UMI for donor 2); n indicates the number of functional clonotypes retained per replica. Self-comparisons (F2 = 1 by definition) are shown in grey; all heatmaps share a common color scale.

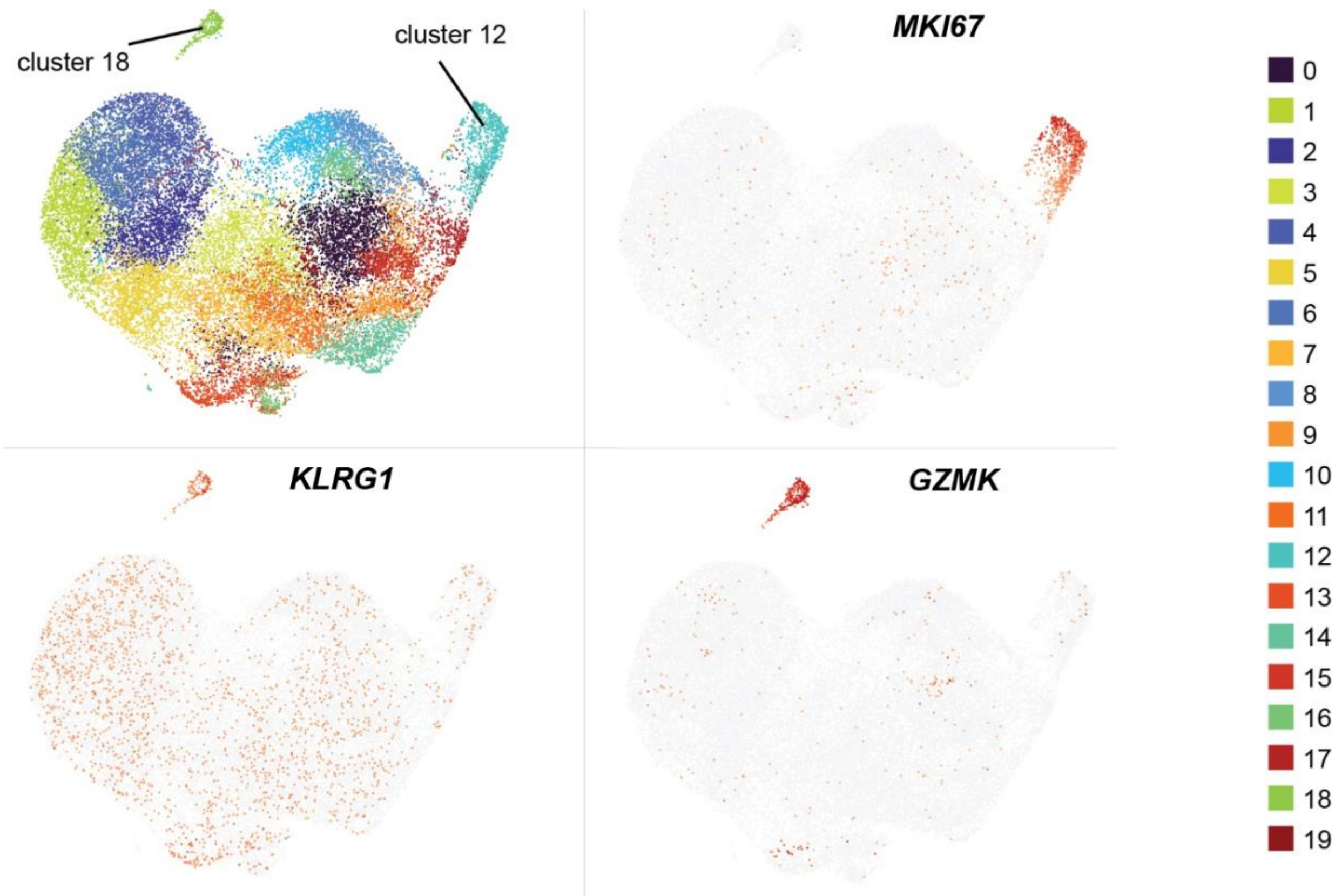

Supplementary Figure 2. Tonsillar follicular CD4+ T cell single-cell cluster analysis.

UMAP embedding of 20 s.c. clusters (left) with expression of *MKI67*, *KLRG1*, and *GZMK*. Cluster 12 (proliferating cells, *MKI67*$^{+}$) and cluster 18 (cytotoxic cells, *KLRG1*$^{+}$ *GZMK*$^{+}$). Data combined from 2 donors.

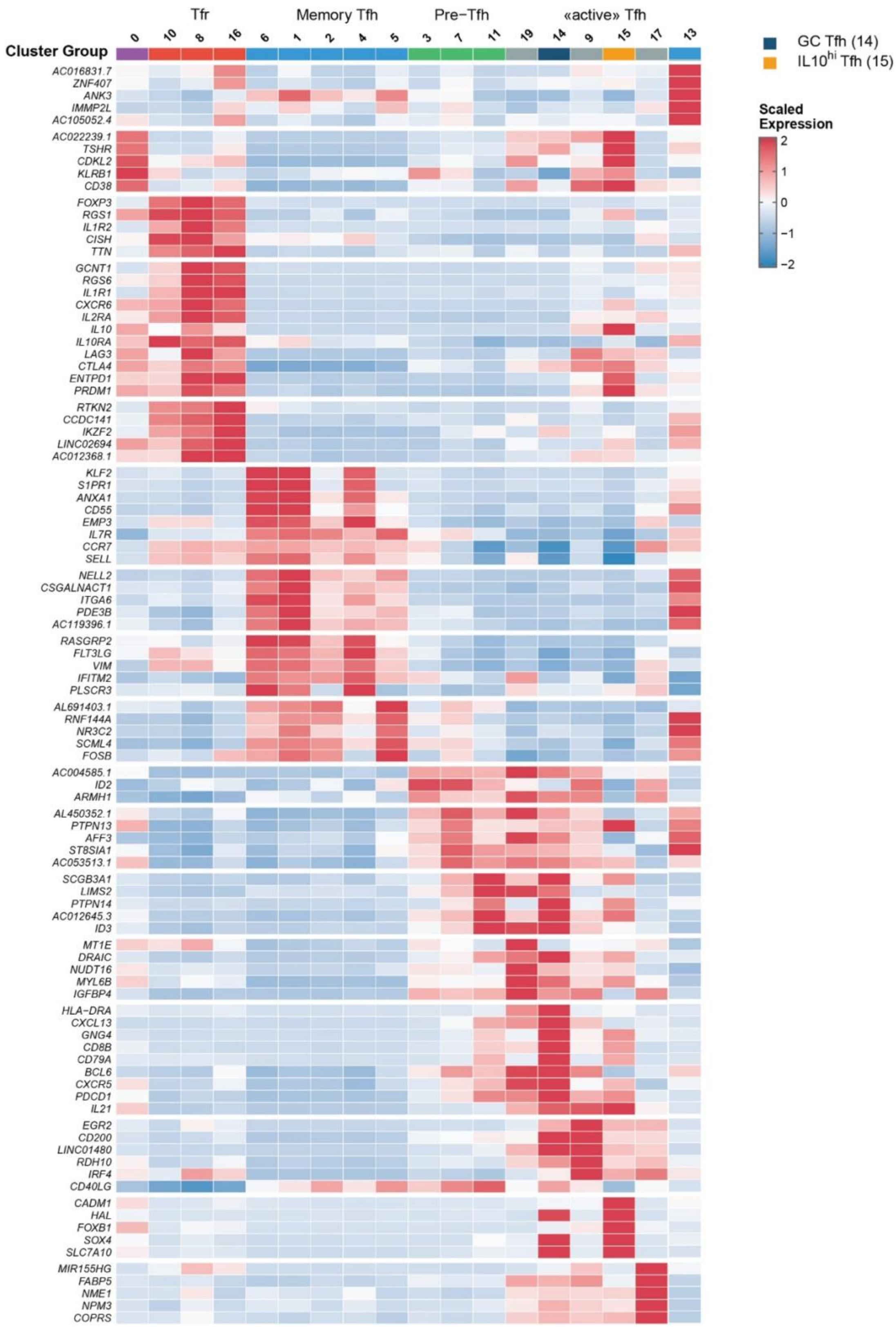

Supplementary Figure 3. Top differentially expressed genes across tonsillar follicular single-cell CD4$^+$ T cell clusters. Heatmap shows top 5 unique differentially expressed genes per cluster supplemented with manually added marker genes: *BCL6, CXCR5, PDCD1, IL21, CD40LG, IL7R,*

CCR7, S1PR1, KLF2, SELL, CD38, KLRB1, IL10, IL10RA, IL2RA, LAG3, CTLA4, ENTPD1, FOXP3, PRDM1. Color indicates median log-normalized gene expression z-scored across clusters per gene, capped at ±2. Clusters 12 and 18 excluded. Data combined from 2 donors.

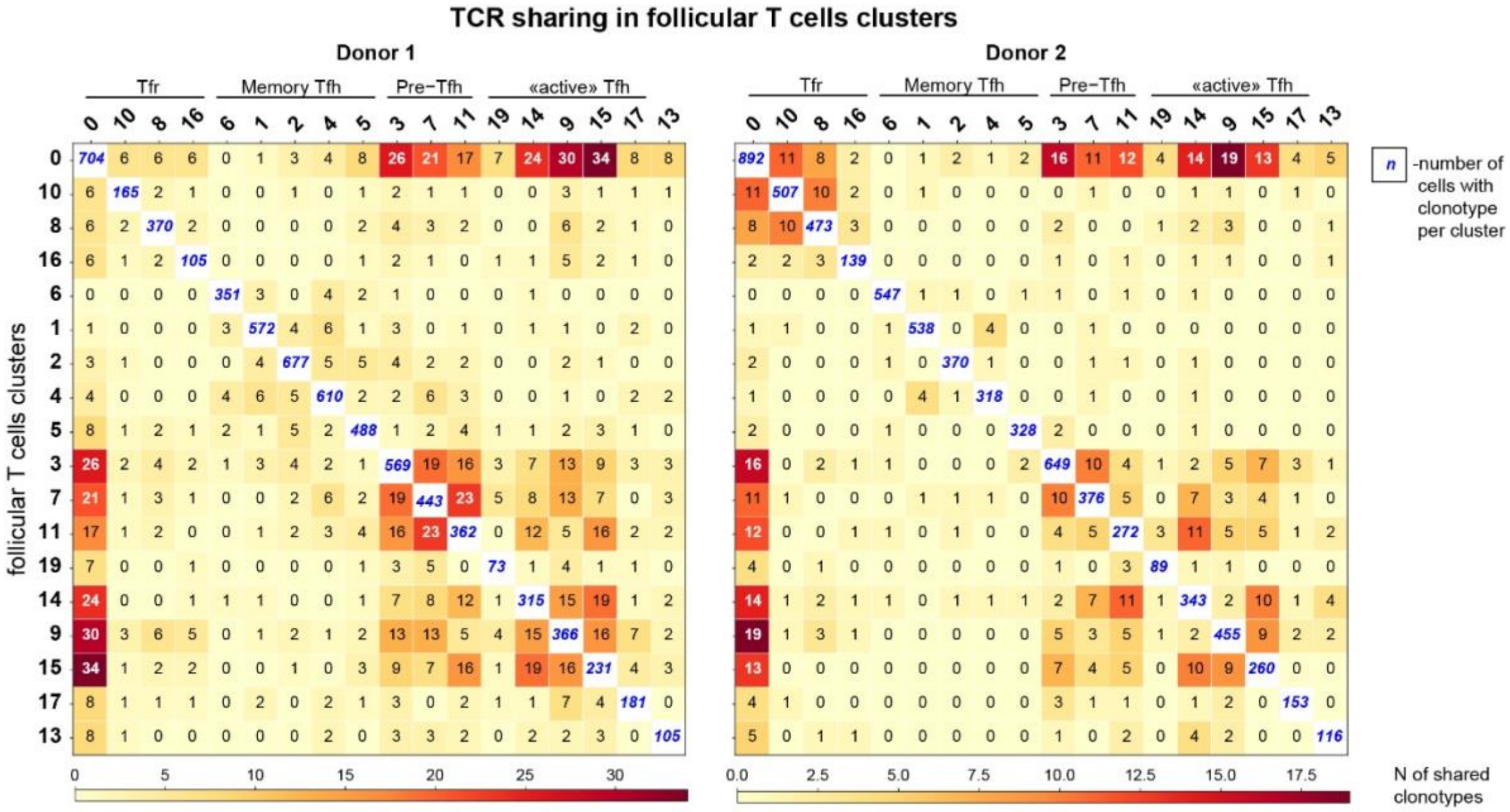

Supplementary Figure 4. Heatmaps of shared TCRβ clonotypes between each pair of single-cell clusters for two donors.

n indicates the number of cells per cluster with a detected clonotype.

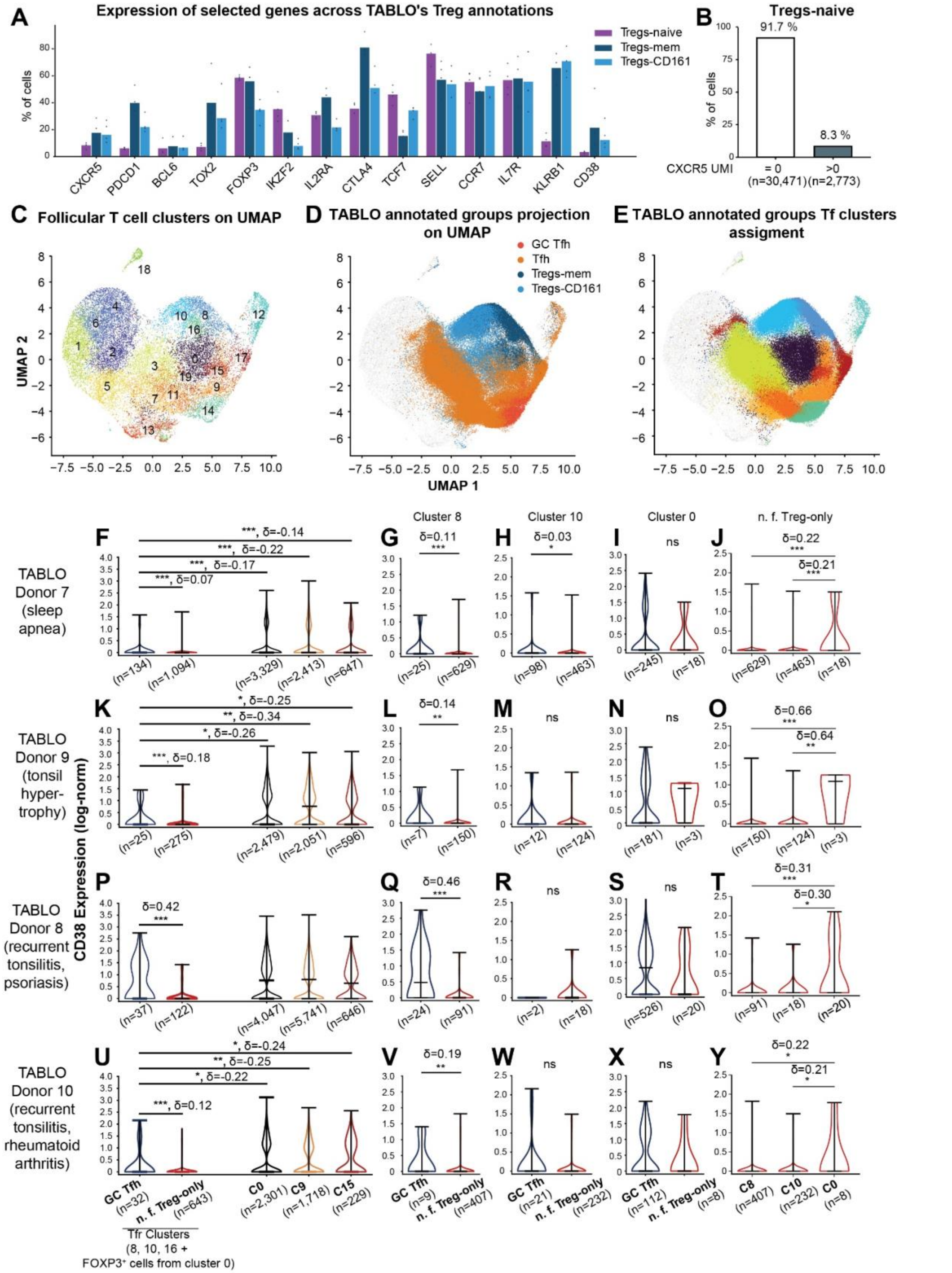

Supplementary Figure 5. Validation of *CD38* expression in iTfr/nTfr and active Tfh cells in

distinct tonsillar follicular T cell clusters in an independent adult cohort (TABLO atlas). (A) Percentage of cells expressing *CXCR5*, *PDCD1* and other indicated genes across the three Treg annotations of the single-cell TABLO atlas (naive, memory and CD161+ Tregs); bars show the mean of 4 donors, dots individual donors. **(B)** Fraction of TABLO naive Treg cells with no detected *CXCR5* UMI, which were used as the n.f. Treg reference repertoire. **(C)** UMAP of the 20 single-cell clusters of tonsillar follicular T cells of this study, used as the projection reference. **(D, E)** TABLO tonsillar memory and CD161+ Treg and Tfh cells projected onto the reference UMAP, colored by TABLO annotation group **(D)** and by the reference cluster they were assigned to **(E)**. **(F–Y)** CD38 expression (log-normalized) in cells of the Tfr pool (reference clusters 8, 10, 16 and FOXP3+ cells of cluster 0) of each TABLO donor: donor 7 **(F–J)**, donor 9 **(K–O)**, donor 8 **(P–T)** and donor 10 **(U–Y)**. For each donor, the first four panels show cells categorized by paired αβ clonotype sharing with GC Tfh or with n.f. Tregs only: the whole Tfr pool, with CD38 expression in Tf clusters 0, 9 and 15 given for reference **(F, K, P, U)**, followed by cluster 8 **(G, L, Q, V)**, cluster 10 **(H, M, R, W)** and cluster 0 **(I, N, S, X)**. The fifth panel of each row **(J, O, T, Y)** shows CD38 expression within the n.f. Treg-only category alone, comparing the cells assigned to clusters 8, 10 and 0. Violins show the distribution of CD38 expression with the median and the extreme values; n indicates the number of cells per category. Statistical comparisons are two-sided Mann–Whitney U tests with Benjamini-Hochberg adjustment for panels **F, K, P, U, J, O, T, Y**. Reference groups were GC Tfh clonotype-sharing Tfr cells (in **F, K, P, U**) and n.f. Tregs clonotype-sharing cells in cluster 0 (**in J, O, T, Y**). δ is Cliff's δ; *, $p \leq 0.05$; **, $p \leq 0.01$; ***, $p \leq 0.001$; ns, not significant.

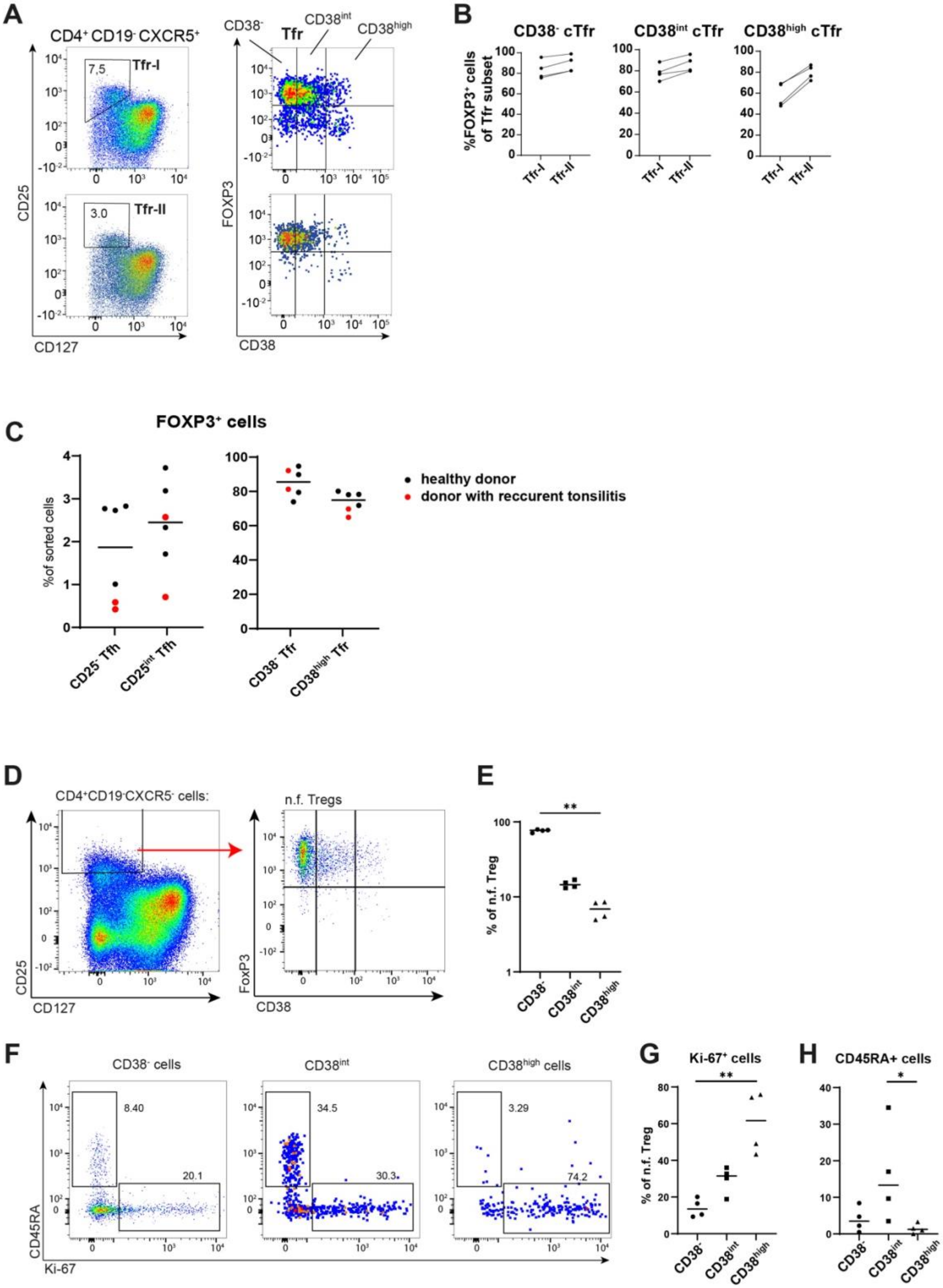

Supplementary Figure 6. Expression of FOXP3+ in Tfh/Tfr subsets and CD38, CD45RA and KI67 on n.f. Treg cells from peripheral blood. (**A, B**) Sensitivity analysis of intracellular FOXP3 expression in CD38^{-}, CD38^{int}, CD38^{high} Tfr subsets using the gates Tfr-I and Tfr-II. The gating

strategy (in **A**) and % of cells expressing FOXP3 (in **B**). (**C**) Percent of FOXP3+ cells in $CD25^-$ and $CD25^{int}$ Tfh, and $CD38^-$ and $CD38^{high}$ Tfr subsets gated as shown in Fig. **5A**. (**D, E**) $CD38^{high}$, $CD38^{int}$ and $CD38^{low}$ n.f. Treg cells gating strategy (**D**) and frequencies (**E**). (**F–H**) Intracellular KI67 and surface CD45RA expression on $CD38^{high}$, $CD38^{int}$ and $CD38^{low}$ n.f. Tregs. (**F**) Gating strategy. (**G, H**) Percentage of $KI67^+$ (**G**) and $CD45RA^+$ (**H**) cells of n.f. Tregs. Analysis was performed on PBMC from healthy donors (n=4, black dots) and from donors with recurrent tonsilitis (n=2, red dots, in **C**). The Kruskal-Wallis test with Dunn`s correction for multiple comparisons was used for statistical description of n.f.Treg cell composition (**E, G, H**). *, $p \leq 0.05$; **, $p \leq 0.002$.

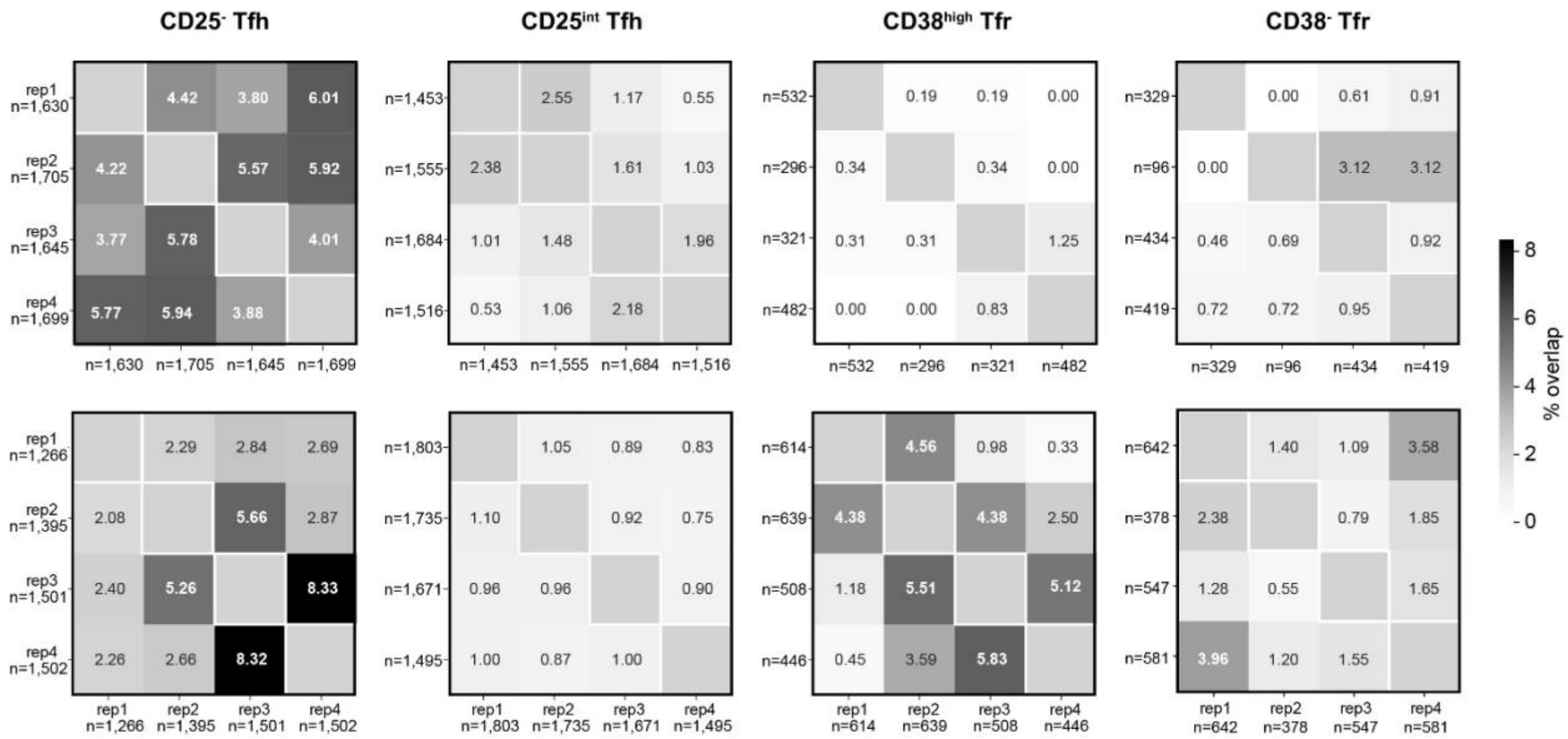

Supplementary Figure 7. Peripheral blood follicular T cell subsets analysis. Pairwise TCRβ clonotypic overlap (percentage) between the replicas within each follicular T cell subpopulation from the peripheral blood of donors with recurrent tonsilitis 1 (top row) and 2 (bottom row). n indicates functional clonotype count per replica.

2 Other Supplementary Data

2.1 Supplementary Movie 1.

Two- to three-dimensional UMAP transition of tonsillar follicular $CD4^+$ T cells. 3D animation of the UMAP embedding of $CD4^+CD19^-CXCR5^+$ T cells from two donors profiled by 10X 5′ scRNA-seq (cluster 12 and 18 excluded). The animation opens on the canonical 2D UMAP layout (matching Fig. 2A) and morphs into the corresponding 3D UMAP, illustrating proximal position of the early memory follicular T cell cluster 13 (orange) and Tfr cluster 16 (light green).

2.2 Supplementary Tables (excel file)

Tab. S1. Patient cohort information table.

Tab. S2. List of antibodies used in the study.

Tab. S3. Bulk TCR-seq sequencing and clonotype statistics: per-sample cell counts, read counts, UMI and clonotype metrics for the circulating blood populations ($CD25^-$ Tfh, CD25 int Tfh, $CD38^-$ Tfr, CD38 high Tfr) and tonsillar populations (GC Tfh, n.f. Treg) from donors 1 and 2 (4 replicates per population).

Tab. S4. Single-cell sequencing and TCR repertoire quality-control metrics. Single-cell sequencing and TCR repertoire QC metrics for the two tonsillar samples profiled by 10X Chromium 5′scRNA-seq with paired V(D)J scTCR-seq. Metrics extracted from the Cell Ranger v9.0.0 multi web summary "Estimated number of cells (productive TCR contigs)" denotes the number of cell barcodes with at least one productive V(D)J contig recovered.

2.3 Supplementary Data (excel file). Raw data and statistical analysis.
